# Associations between diet composition, dietary pattern, and weight outcomes after bariatric surgery: a systematic review

**DOI:** 10.1038/s41366-023-01333-1

**Published:** 2023-07-06

**Authors:** H. C. Cheung, E. Strodl, J. Musial, H. L. MacLaughlin, A. Byrnes, C-A. Lewis, L. J. Ross

**Affiliations:** 1 Queensland University of Technology, School of Exercise and Nutrition Sciences, Faculty of Health, Brisbane, QLD Australia; 2 Queensland University of Technology, School of Psychology and Counselling, Faculty of Health, Brisbane, QLD Australia; 3 Department of Nutrition and Dietetics, Clinical Support Services, Princess Alexandra Hospital, Brisbane, QLD Australia; 4 Nutrition Research Collaborative, Department of Dietetics and Food Service, Royal Brisbane and Women’s Hospital, Herston, Brisbane, QLD Australia

**Keywords:** Nutrition, Obesity, Weight management

## Abstract

**Introduction:**

Literature describing the impact of dietary intake on weight outcomes after bariatric surgery has not been synthesized. This study aimed to synthesize the evidence regarding any association between diet composition and weight outcomes post-bariatric surgery.

**Methods:**

CINAHL, Cochrane, Embase, MEDLINE and Scopus were searched for adult studies up to June 2021 that assessed any association between dietary intakes (≥1-macronutrient, food group, or dietary pattern) and weight outcomes at 12-months or longer after bariatric surgery. Risk of bias and quality assessments were conducted using the Scottish Intercollegiate Guidelines Network checklists and the NHMRC’s Level of Evidence and Grades for Recommendations. Study findings were presented according to the time of post-surgery dietary intake assessment (≤12months, between 12 and 24 months, ≥24months).

**Results:**

5923 articles were identified, 260 were retrieved for full text screening, and 36 were eligible for inclusion (9 interventional including five randomized-controlled trials, and 27 observational cohort studies; sample sizes: 20–1610; total sample: 5065; follow-up periods: 1 year–12 years; level of evidence: II to IV, risk of bias: low to high). Findings on the association between long-term weight outcomes and dietary composition up to 24-months were mixed. After 24-months, studies consistently suggested no significant associations between weight loss and macronutrient composition or core food group patterns, or between carbohydrate, protein or food group patterns and weight recurrence. A single cohort study reported a weak association between diet quality score and weight-recurrence after 24-months.

**Conclusion:**

There was no strong evidence to support significant associations between diet composition and weight outcomes post-bariatric surgery. The heterogeneity in study design and quality may reduce generalizability to external populations. Individualized dietary recommendations may be useful to support long-term post-surgery weight outcomes. More studies are needed to define and measure diet quality in this patient cohort.

**Registration:**

PROSPERO (CRD42021264120)

## Introduction

Bariatric surgery is considered the gold-standard treatment for inducing significant weight loss, which can alleviate obesity-related complications in people with severe obesity [1]. In recent years, the demand for bariatric surgery has increased with the rising prevalence of obesity [2]. From 2011 to 2019, the total number of bariatric surgeries performed worldwide rose from 158,000 to 256,000 [2]. However, weight non-response (insufficient weight loss) or weight recurrence (weight regain) are reported in as many as 1 in 2 patients at 2 years, and 3 in 5 patients at 12 years after surgery [3, 4]. These are associated with reduced quality of life, re-occurrence of obesity-related complications, deteriorated health, and ultimately, escalated health care costs and mortality [3–7].

Previous studies have explored the factors contributing to weight non-response or significant weight recurrence following bariatric surgery, and identified patients’ post-operative diet to be a key modifiable determinant of weight status post-surgery [5, 8]. In addition, Zarshenas and colleagues’ systematic review reported poor diet quality among patients at least one year after bariatric surgery [9]. These findings highlighted the role of dietary intakes in the management of weight after bariatric surgery, and the importance of nutritional interventions to improve the long-term diets of patients post-surgery.

At present, nutritional management guidelines for patients after bariatric surgery either focus on the diet texture progression within the first month post-operatively, or have based their overall diet recommendations, after texture progression (10–35% or >60 g/d protein, 30–70% or >130 g/d carbohydrates, 20–35% fats, and 5 serves of vegetables a day), on extrapolated evidence from non-surgical populations and/or small studies with weak evidence [10–12]. As bariatric surgery leads to significant changes in patients’ anatomy, physiology, and tolerance of specific foods and food volumes, dietary advice intended for the general population may not be suitable for patients post-bariatric surgery [13–16]. Depending on individual needs, stomach capacity, surgical outcomes and time after surgery, there may be a change in macronutrient requirements over-time. More evidence is needed to drive consensus and inform dietary recommendations for the medium to long-term post-surgery [9].

Over the past decade, there have been several studies examining the potential influence of dietary intake on weight loss and/or weight recurrence post-bariatric surgery. Therefore, the aim of this systematic review was to synthesize the available evidence regarding associations between post-surgery dietary intake (macronutrient composition and food patterns) and weight outcomes at least one year and longer after bariatric surgery.

## Methods

The protocol for this review is registered in PROSPERO (CRD42021264120) and was conducted and reported as per the Preferred Reporting Items for Systematic Reviews and Meta-Analysis (PRISMA) checklist [17].

### Search strategy

A systematic search was conducted in electronic databases: CINAHL, Cochrane, Embase, MEDLINE and Scopus for human studies published in English language from all years up to and including June 2021. The basic search strategy was (post OR after OR following) “Bariatric surgery” OR “weight loss surgery” OR gastric bypass” OR “gastric sleeve” OR “sleeve gastrectomy” AND diet* OR nutrition* OR eat* OR macronutrient* OR ‘postoperative diet’. The full search strategy and subject headings used for each database are available in Supplementary Material 1.

### Record screening and eligibility criteria

Title and abstract screening were conducted by HC. Full text screening was completed by members of the research team independently in groups of two (Group 1: HC and LR, Group 2: HC and ES, Group 3: HM and JM) using systematic review software, Rayyan (Rayyan Systems Inc., Cambridge) [18]. Any uncertainties around study inclusion were raised to the research team for discussion until consensus was reached. Eligible study designs were systematic reviews, meta-analyses, interventional and cohort studies of adults (18 years of age or above) without pre-existing life-threatening conditions who received bariatric surgery (any type). Outcomes included at least one post-surgery dietary variable (reported at least 1 macronutrient, food group or dietary pattern) that was compared to any post-surgery weight outcome(s). Studies were included if their analyses involved: (1) weight outcomes in response to a prescribed diet; (2) a comparison of weight change between participant groups and their concurrent diet; and/or (3) correlation analysis of association between weight outcomes and diet. Studies were excluded if: (i) any participants were pregnant or breastfeeding (ii) the participants were inpatients, (iii)a post-surgery progression diet (i.e. texture modified or less than 1 month post-surgery), (iv) measures of adherence to a specific diet or diet preferences without indicating the exact diet being followed, or (v) if intake analysis were limited to energy, micronutrients, test meals, single meals, or supplements only.

### Data extraction

Data extraction was performed by HC and cross-checked by LR. Information extracted included country of publication, participant characteristics including pre- and post-surgery health parameters, assessment timelines, dietary assessment method, dietary intakes (energy, macronutrients, food/dietary patterns) and weight outcomes at any timepoint: excess weight loss, odds of reaching >50% excess weight loss, total weight loss, average monthly weight loss, initial weight loss, BMI loss, risk of obesity remission i.e. BMI < 30 kg/m^2^, presence of weight recurrence as defined by the study authors as exceeding a nominated percentage of weight gain after nadir weight (lowest weight post-surgery), odds of weight recurrence, risk of weight recurrence. Key findings were those regarding any association between dietary intake variables and weight outcome(s), and/or comparisons between intervention and control groups or groups of participants achieving/not achieving pre-defined weight outcome(s) and dietary intake. Statistical analysis results reported in studies were extracted, including correlation co-efficient, odds ratio, hazard ratio and 95% Confidence Interval and interpreted according to conventional standards established by Cohen [19].

### Quality assessment

Individual studies were matched to the Australian National Health and Medical Research Council (NHMRC) levels of evidence and grades for recommendation guidelines depending on study design [20]. Studies were first assigned a level based on the potential of the study design to adequately answer the defined research question(s): with level I being the highest level of evidence, followed by II, III-1, III-2, III-3, and IV (lowest) [20]. The Scottish Intercollegiate Guidelines Network (SIGN) Risk of Bias checklists for cohort, case-control and controlled trial study designs were then used to determine the risk of bias (low risk, acceptable risk, and high risk) [21]. The level of evidence and risk of bias of each study were assessed by HC and cross-checked by LR independently with blinding.

### Data synthesis

To synthesize reporting differences between studies, dietary and outcome data were treated as follows: Intake assessments reported over different timeframes were grouped and presented as three main post-surgery time-dependent categories: up to one year (≤12 months); between one and two years ( > 12 to <24-months); and two years or longer (≥24 months). Studies were then further categorised within each assessment timeframe according to the dietary variables reported (macronutrient composition or food pattern). Within these dietary categories, all associated weight outcomes were included regardless of follow-up timeframes (equivalent or longer than the dietary timeframes) and grouped as two main outcome categories: (1) weight loss (including excess weight loss (EWL), initial weight loss (IWL), total weight loss (TWL), body mass index (BMI) loss), and obesity remission (i.e. reaching a BMI of <30 kg/m^2^); or (2) weight recurrence measures of odds ratio, hazard ratio, or presence of weight recurrence from nadir weight that had exceeded a study-specified percentage.

### Data analysis

The bodies of evidence regarding the associations between post-surgery weight outcomes and individual macronutrients and food patterns were assessed and graded using the NHMRC Guidelines [20]. In accordance with these guidelines, the bodies of evidence were assessed based on five components, and each component has been graded based on a set of standard criteria [20].

Recommendations were then deduced from these bodies of evidence and graded based on the combined gradings from each graded component. The possible grades for recommendations were: Grade A (body of evidence can be trusted to guide practice); Grade B (body of evidence can be trusted to guide practice in most situations); Grade C (body of evidence provides some support for recommendation(s) but care should be taken in its application); and Grade D (body of evidence is weak, and recommendation must be applied with caution). The grading of evidence was conducted by HC, cross-checked by LR, then reviewed and achieved consensus with ES, JM, AB, and CL.

## Results

**Fig. 1:**
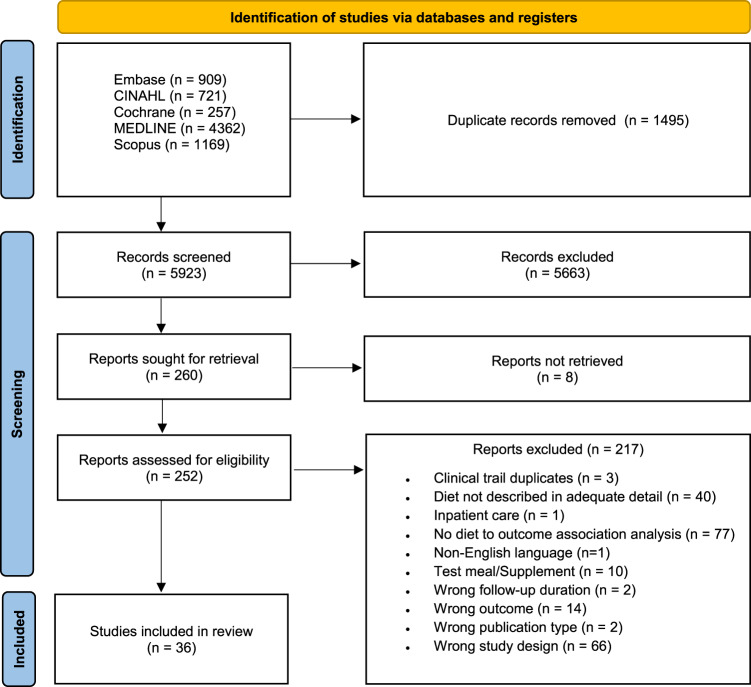
PRISMA Flow Diagram. Summary of the identified, screened, and included studies from databases and registers.

The screening process of this review is outlined in Fig. 1. A total of 5923 records were identified and title/abstract screened after the removal of 1495 duplicates. A total of 260 records were retrieved for full text screening, and 36 papers were included in this review. Reasons for exclusion were listed in Fig. 1.

### Study characteristics

The key characteristics of the 36 included studies are summarized in Table 1 [22–57]. The retrieved study designs included nine intervention studies (5 randomised controlled trials, 3 non-randomized controlled trials, 1 pre-post-intervention) [29, 32, 34, 36, 37, 47, 52, 53, 55]. Interventions delivered typically consisted of lifestyle modifications with or without a prescribed diet plan, where participants’ dietary intakes (macronutrient composition and/or food group pattern) were recorded and compared against pre-intervention values and/or between study intervention or control groups. In total, 27 observational cohort studies (8 prospective and 19 retrospective) were included [22–28, 30, 31, 33, 35, 38–46, 48–51, 54, 56, 57]. These studies compared the diets of participants grouped according to their weight status, and/or conducted direct tests of association between dietary variable(s) and weight outcome(s).

**Table 1:** Characteristics of included studies.

Author, Date (Country)	Study Design	Study Grading and Risk of Bias	Comparison groups or tests of association	Surgery type (%)	Dietary intake assessment tool (Monitoring intake)	Number of participants	Drop-out or exclusion (%)	Participant age (years)	Gender distribution (%female)	Pre-surgery insulin resistant / T2DM (%)	Time of post-surgery outcomes
Alvarez et al., 2017 [22] (Chile)	Retrospective cross-sectional case-control	III-3, Low	1. Weight regain (%) at <50^th^ percentile 2. Weight regain (%) at >50^th^ percentile	SG (100%)	Food Frequency Questionnaire (Nil)	1.: 20 2.: 20	n/a	1.: Median 41.5 (IQR 33.3–58.5) 2.: Median 39.5 (IQR 37–48)	80%	T2DM: NR Insulin resistant: 52.5	1. Median 39 (IQR 33–42.5) months 2. Median 36.5 (IQR 34–41) months
Amundsen et al., 2017 [23] (Norway)	Retrospective cross-sectional case-control	III-3, Low	Comparison 1 1. Excess weight loss ≥50% 2. Excess weight loss <50% Comparison 2 1. Weight regain ≤15% 2. Weight regain >15%	GB (100%)	Food Frequency Questionnaire (Nil)	Comparison 1 1.: 11 2.: 36 Comparison 2 1.: 27 2.: 22	n/a	Comparison 1: 1.: 51.5 ± 7.5 2.: 45.5 ± 7.2 Comparison 2 1.: 47.1 ± 8.2 2.: 46.5 ± 6.9	Comparison 1: 1.: 100% 2.: 81.6% Comparison 2 1.: 88.9% 2.: 81%	T2DM: NR	Comparison 1 1.: 53.9 ± 20.4 months 2.: 59.6 ± 26.0 months Comparison 2 1.: 54.2 ± 21.9 months 2.: 63.4 ± 27.6 months
Bobbioni-Harsch et al., 2002 [24] (Switzerland)	Prospective Observational	II, Acceptable	Associations with excess weight loss and total weight loss	RYGB (100%)	3-day Food Record & Diet recall (Nil)	50	NR	38.4 ± 1.4	100%	T2DM: NR	12 months
Cadena-Obando et al., 2020 [25] (Mexico)	Retrospective cross-sectional Observational	III-3, Low	1. Excess weight loss ≥50% 2. Excess weight loss <50%	RYGB (38%), OAGB (49%), SG (13%)	24 h Recall (Nil)	1.: 104 2.: 26	n/a	1.: 46 ± 9** 2.: 52 ± 8	1.: 80% 2.: 81%	T2DM: 26%	12 months
Chou et al., 2017 [26] (Taiwan)	Retrospective case-control	III-3, Acceptable	Associations with total weight loss; 1. Weight regain <25% 2. Weight regain ≥25%	SG (100%)	24 h Recall & Food Frequency Questionnaire (Nil)	1.: 28 2.: 9	7.5%	33.5 ± 9.7	75%	T2DM: NR	60 months
Dagan et al., 2017 [27] (Israel)	Prospective case-control	III-2, Acceptable	1. Excess weight loss ≥60% 2. Excess weight loss <60%	SG (100%)	3-day Food Record (Nil)	1.: 66 2.: 11	23%	43.1 ± 9.3	57.1%	T2DM: NR	12 months
da Silva et al., 2010 [28] (Brazil)	Retrospective cross-sectional case-control	III-3, Low	Odds of weight regain ≥10%; 1. Weight regain <10% 2. Weight regain ≥10%	RYGB (100%)	2 × 24 h Recalls & Healthy Eating Index (Adapted for the Brazilian population) (Nil)	1.: 61 2.: 19	n/a	1.: Median 46.0 (IQR 30–62) 2.: Median 43.0 (IQR 23–63)	1.: 88.5% 2.: 89.5%	T2DM: NR	1.: Median 39.0 (IQR 29–49)*** months 2.: Median 56.5 (IQR 22.5–90.5) months
Dodsworth et al., 2012 [29] (Australia)	Pseudo-Randomized controlled trial	III-1, Acceptable	Intervention: 6-month prescribed protein-enriched diet (960–1400 kcal/day, 40% carbohydrate, 30% protein, 25% fat). Control: Usual care; Associations with BMI, excess weight loss and total weight loss	LAGB (100%)	3-day Food Record & 24 hr recall (Nil)	Intervention: 24 Control: 23	Intervention: 21% Control: 39%	Intervention: 44.9 ± 11.3 Control: 44.0 ± 10.0	Intervention: 83.3% Control: 78.3%	T2DM: NR	12 months
Faria et al., 2009 [30] (Brazil)	Retrospective observational	IV, Acceptable	Associations with average monthly weight loss	RYGB (100%)	4-day Food Record (Nil)	89	Diet analysis: 40%	36.8 ± 10.7	80%	T2DM: NR	NR (Included both 6–12 and >12 months patients)
Faria et al., 2009 [31] (Brazil)	Retrospective cross-sectional cohort	III-3, High	1. <150 kcal of sweets/snacks between main meals. Highest excess weight loss (%) in cohort (*p* < 0.05 with 3. only). 2. ≥150 kcal of sweets between main meals. 3. ≥150 kcal of snacks between main meals.	RYGB (100%)	4-day Food Record (Nil)	1.: 34 2.: 27 3.: 14	n/a	36.8 ± 10.7	80%	T2DM: NR	23 ± 10.3 months
Faria et al., 2010 [32] (Brazil)	Pre-post interventional	IV, High	Intervention: 3-month prescribed high protein diet (1400 ± 180 kcal/day, 45% carbohydrate, 35% protein, 20% fat, 3 serves/day dairy). Control: n/a	RYGB (100%)	7-day Food Record (Nil)	30	n/a	36 ± 11	87%	T2DM: NR	4 ± 1.4 years
Freire et al., 2011 [33] (Brazil)	Retrospective cross-sectional case-control	III-3, Acceptable	Associations with excess weight loss; 1. Weight regain <2% 2. Weight regain ≥2%	RYGB (100%)	24 h Recall (Nil)	1.: 44 2.: 56	n/a	1.: 46.5 ± 8.9 2.: 43.9 ± 10.5	1.: 77.3% 2.: 89.3%	T2DM: NR	45.5 ± 32.6 months
Gallé ´ et al., 2020 [34] (Italy)	Non-randomised controlled trial	III-2, Acceptable	Intervention: 12-month exercise, motivational, and nutritional program. Control: Received routine medical examinations only.	Intervention: SG (64.3%), LAGB (35.7%) Control: SG (54.8%), LAGB (45.2%)	7-day Food Record (Nil)	Intervention: 28 Control: 42	Intervention: 7% Control: 19%	Intervention: 38.2 ± 8.7 Control: 40.2 ± 9.5	Intervention: 85.7% Control: 71.4%	T2DM: NR	12 months
Iossa et al., 2020 [35] (Italy)	Retrospective case-control	III-3, Low	Associations with excess weight loss, risk of risk of excess weight loss <50%, risk of weight regain >25%; 1. Excess weight loss >50% at first year + <25% regain of excess weight 2. Excess weight loss <50% at first year 3. >25% regain of excess weight loss in absence of surgical complications	SG (100%)	7-day Food Record (Nil)	1.: 58 2.: 4 3.: 24	27%	45 ± 10	1.: 78% 2.: 75% 3.: 79%	T2DM: 11.6%	7 years
Jassil et al., 2015 [36] (United Kingdom)	Non-randomised controlled trial	III-2, Acceptable	Intervention: 8-week exercise training, and group lifestyle and nutritional behavioural change sessions, at 3–6 months. Control: Standard post-surgical care and follow-ups.	RYGB (25%), SG (75%)	Food Frequency Questionnaire (Nil)	Intervention: 8 Control: 16	20%	Intervention: 44.8 ± 8.2 Control: 45.5 ± 8.3	100%	T2DM: 37.5%	12 months
Kalarchian et al., 2016 [37] (United States)	Randomized controlled trial	II, Acceptable	Intervention: 4-month prescribed diet (4 serve/day vegetables, 2–4 serves/day meat, and 1–2 serves/day low glycaemic index grains) with home delivered, portion-controlled meals + usual care. Control: Usual care.	RYGB (100%)	24 h Recall (Nil)	Intervention: 20 Control: 20	Primary analysis with full sample: 0%; Secondary analysis excluding outlier: 5%	Intervention: 47.8 ± 12.1 Control: 46.0 ± 10.2	Intervention: 85% Control: 85%	T2DM: NR	16 and 18 months
Kanerva et al., 2017 [38] (Sweden)	Prospective Matched Non-randomised cohort	II, Low	Associations with initial weight loss between men and women, according to changes in intakes from 0-6 months.	GB (15.4%), gastric banding (16.1%), VBG (68.5%)	Semi-quantitative Food Frequency Questionnaire (Nil)	1610	26%	47.3 ± 6.1 female 47.3 ± 5.8 male	70%	T2DM: NR	10 years
Kruseman et al., 2010 [39] (Switzerland)	Retrospective cross-sectional case-control	III-3, Low	1. Excess weight loss ≥50% 2. Excess weight loss <50%	RYGB (100%)	4-day Food Record (Nil)	1.: 47 2.: 33	n/a	1.: 37 ± 10* 2.: 44 ± 9	100%	T2DM: NR	1 and 8 years
Lim et al., 2020 [40] (Korea)	Retrospective cross-sectional case-control	III-3, Low	Associations with excess weight loss, odds of excess weight loss ≥50%; 1. Excess weight loss ≥50% 2. Excess weight loss <50%; 12-month intake cut-offs for reaching excess weight loss ≥50%	RYGB (77.2%), SG (22.8%)	3-day Food Record (Nil)	1.: 127 2.: 62	n/a	34.6 ± 10.7	71.4%	T2DM: 18%	12 months
Lindroos et al., 1996 [41] (Sweden)	Prospective observational	IV, Low	Associations with weight change; TWL between participants with highest vs lowest quartiles of intakes	VBG (72%), GB (28%)	Food Frequency Questionnaire (Nil)	375	3%	46 ± 6	66%	T2DM: NR	24 months
Masood et al., 2019 [42] (Saudi Arabia)	Retrospective cross-sectional case-control	III-3, High	1. Weight regain <15% 2. Weight regain ≥15%	NR	Purpose designed Diet and Behaviour Questionnaire (Nil)	1.: 29 2.: 21	n/a	1.: 41 ± 9.9 2.: 41 ± 7.5	1.: 65.5% 2.: 42.9%	T2DM: NR	18 months
Moizé, V. et al., 2013 [43] (Spain)	Prospective observational	II, Low	Associations with EWL.	RYGB (83%), SG (17%)	3-day Food Record & 24 h recall (Nil)	SG: 61 RYGB: 294	24 months: SG: 16%, RYGB: 12%. 36 months: SG: 41%, RYGB: 31%. 60 months: SG: 66%, RYGB: 53%.	SG: 46.4 ± 11.6 RYGB: 45.2 ± 10.6	SG: 67.2% RYGB: 77%	T2DM: NR	Median 48 months (IQR 24-60)
Novais et al., 2012 [44] (Brazil)	Retrospective cross-sectional case-control	III-3, Acceptable	1. Excess weight loss ≥75% 2. Excess weight loss 50–75%3. Excess weight loss <50%	RYGB (100%)	2 × 24 h recalls (Nil)	1.: 51 2.: 68 3.: 22	n/a	1 + 2: 44 ± 9 3.: 48 ± 10	100%	T2DM: NR	3.9 ± 1.4 years
Ortega et al., 2012 [45] (Spain)	Retrospective observational	IV, Acceptable	Associations with BMI loss.	RYGB (100%)	72 h recall	107	18% for diet analysis	41.8 ± 9.8	79%	T2DM: NR	3 ± 1.8 years
Palacio et al., 2020 [46] (Chile)	Retrospective cross-sectional case-control	III-3, Acceptable	Associations with odds of weight regain ≥15%; 1. Weight regain <15% 2. Weight regain ≥15%	1.: RYGB (58%) & SG (42%) 2.: RYGB (42.6%) & SG (57.4%)	3 × 24 h recalls (Nil)	1.: 50 2.: 54	n/a	1.: 40.8 ± 10.1 2.: 43.1 ± 10.1	1.: 70% 2.: 77.8%	T2DM: NR	24 months
Papalazarou et al., 2010 [47] (Greece)	Randomised controlled trial	II, High	Intervention: 3-years person-centred lifestyle behaviour change program + usual care. Control: Usual care.	VBG (100%)	7-day Food Record (Nil)	30	NR	Intervention: 32.7 ± 1.6 Control: 33.4 ± 2.0	Intervention: 100% Control: 100%	T2DM: NR	1 and 3 years
Pinto et al., 2019 [48] (Brazil)	Prospective observational	II, Low	Associations with risk of obesity remission (BMI < 30)	RYGB (100%)	3 × 24 h recalls (Nil)	51	2%	39.34 ± 9.38	69%	T2DM: NR	12 months
Raftopoulos et al., 2011 [49] (United States)	Prospective observational	II, Low	Associations with BMI change and excess weight loss	RYGB (100%)	7-day Food Record (Nil)	Complete data: 167 Incomplete data: 126	73% of participants with incomplete data ( = 32.4% of cohort)	Complete data: 42.7 ± 11** Incomplete data: 38.6 ± 10.7	Complete data: 83.8% Incomplete data: 87.3%	T2DM: NR	12 months
Reid et al., 2016 [50] (Canada)	Retrospective cross-sectional case-control	III-3, Low	1. Total weight loss ≥38% 2. Total weight loss ≤30%	RYGB (100%)	3-day Food Record (Nil)	1.: 10 2.: 17	n/a	1.: 54.4 ± 7.6 2.: 51.1 ± 9.6	89%	T2DM: NR	11.15 ± 3.7 years
Ruiz-Lozano et al., 2016 [51] (Spain)	Prospective case-control	III-3, Acceptable	1. Excess weight loss ≥50% at nadir and until last follow-up 2. Excess weight loss <50% at nadir and up to last follow-up 3. Excess weight loss ≥50% at nadir but <50% at last follow-up	1.: RYGB (76.9%), SG (23.1) 2.: RYGB (86.2%), SG (13.8) 3. RYGB (63.8%), SG (36.2%)	4-day Food Record (Nil)	1.: 183 2.: 29 3.: 58	15%	1.: 50.4 ± 11.0** 2.: 57.3 ± 8.7 3.: 54.1 ± 11.5	1.: 81.9%* 2.: 86.2% 3.: 65.5%	T2DM: NR	Nadir (18–24 months) and 60 months
Sarwer et al., 2012 [52] (United States)	Randomised controlled trial	II, High	Intervention: 4-month dietary counselling sessions + usual care. Control: Usual care.	RYGB & LAGB (NR)	Food Frequency Questionnaire (Nil)	Intervention: 41 Control: 43	50%, 60.7%, 56% at 12, 18, 24 months respectively.	42 ± 9.9	63%	T2DM: NR	12, 18, 24 months
Schiavo et al., 2017 [53] (Italy)	Randomised controlled trial	II, Acceptable	Intervention: 12-month prescribed protein-enriched diet (1200 kcal/day, 37.3% carbohydrate, 47.7% protein, 15% fat). Control: 12-month prescribed normal-protein diet (1200 kcal/day, 61.7% carbohydrate, 23.3% protein, 15% fat).	SG (100%)	3-day Food Record & 72 h recall (Monitored intakes by comparing results of 3-day food record and 72 h recall)	Intervention: 30 Control: 30	Intervention: 7% Control: 10%	Intervention: 43 ± 5.5 Control: 41 ± 6.2	Intervention: 0% Control: 0%	T2DM: NR	12 months
Schiavo et al., 2018 [54] (Italy)	Retrospective cross-sectional cohort	III-3, High	1. Prescribed low-purine diet (890 kcal/day, 55% carbohydrate, 20% protein, 25% fat, emphasis on low-purine foods). 2. Prescribed normal-purine diet (890 kcal/day, 55% carbohydrate, 20% protein, 25% fat, no emphasis on low-purine foods).	SG (100%)	3-day Food Record & 72 h recall (Monitored intakes by comparing results of 3-day food record and 72 h recall)	1.: 24 2.: 16	n/a	NR	NR	T2DM: NR	12 months
Taus et al., 2017 [55] (Italy)	Non-randomised controlled trial	III-2, High	Intervention: 2-month prescribed ketogenic diet (800 kcal/day, 20% carbohydrate, 40% protein, 40% fat). Control: Prescribed usual care diet 800 kcal/day, 52% carbohydrate, 25% protein, 23% fat + Band calibration (Average 8cc).	LAGB (100%)	NR (Nil)	Intervention: 10 Control: 10	0%	41.42 ± 7.5	75%	T2DM: NR	14 months
Wardé-Kamar et al., 2004 [56] (United States)	Retrospective observational	III-3, High	Associations with excess weight loss; 1. Excess weight loss ≥50% 2. Excess weight loss <50%	RYGB (100%)	24 h Recall (Nil)	1.: 35 2.: 20	Diet analysis: 10%	46 ± 11	93%	T2DM: NR	30 ± 8 months
Yanos et al., 2015 [57] (United States)	Retrospective cross-sectional observational	IV, Acceptable	Associations with risk of weight regain ≥20% or total weight loss at nadir	RYGB (100%)	Bariatric Surgery Self-management Questionnaire (Nil)	97	n/a	56.11 ± 11.26	77.3%	T2DM: NR	8.86 ± 3.59 years

Results were presented in Mean ± Standard Deviation unless otherwise specified.

*p < 0.05, **p < 0.01, ***p < 0.001 for inter-group differences.

*GB* Gastric bypass, *LAGB* Laparoscopic Adjustable Gastric Banding, *n/a* Not applicable, *NR* Not reported by study, *OAGB* One anastomosis gastric bypass, *RYGB* Roux-en-Y gastric bypass, *SG* Sleeve gastrectomy, *T2DM Type 2 Diabetes Mellitus, VBG* Vertical banded gastroplasty.

A range of bariatric surgery procedures were reported among the included studies, with some individual studies reporting more than one type. The most common procedures were Roux en Y gastric bypass (RYGB) (*N* = 22 studies) [24, 25, 28, 30–33, 36, 37, 39, 40, 43–46, 48–52, 56, 57] and sleeve gastrectomy (SG) (*N* = 13 studies) [22, 25–27, 34–36, 40, 43, 46, 51, 53, 54]. Others included laparoscopic adjustable gastric banding (LAGB) (*N* = 4 studies) [29, 34, 52, 55], vertical banded gastroplasty (VBG) (*N* = 3 studies) [38, 41, 47], gastric bypass (did not specify type) (*N* = 3 studies) [23, 38, 41], gastric banding (did not specify type) (*N* = 1 study) [38], and anastomosis gastric bypass (OAGB) (*N* = 1 study) [25]. One study did not specify the type of bariatric surgery involved [42]. Dietary intake assessment tools (from most to least common) were food records (3–7 days), followed by 24-hour recalls, food frequency questionnaires, and other lifestyle or behavior surveys collecting dietary data as part of the tool [22–57]. Sample size ranged from 20 to 1610 participants (total *N* = 5065) with drop-out rate from 0 to 61%. The reported mean age of participants ranged from 32.7 ± 1.6 years to 57.3 ± 8.7 years. The proportion of female participants totaled across all studies was 75% (excluding the single study where gender ratio was not reported) [54]. The majority of studies did not mention the proportion of participants with insulin resistance or type 2 diabetes mellitus (T2DM). Even though some studies did report on the percentage of participants with at least one co-morbidity, they did not specify the exact proportion of participants with T2DM [33, 57]. Among studies that reported on them, the rate of participants with insulin resistance or T2DM mellitus ranged from 11.6%-52.5% [22, 25, 35, 36, 40]. Timepoint of measurement or assessment of post-surgery diets ranged from 6 months to 12 years. Significant post-surgery weight outcomes were reported in all studies from 1 to 12 years follow up.

### Quality assessment

The level of evidence and risk of bias outcomes are reported in Table 1. There were no Level I studies, nine Level II studies [24, 37, 38, 43, 47–49, 52, 53], 22 Level III studies [22, 23, 25–29, 31, 33–36, 39, 40, 42, 44, 46, 50, 51, 54–56], and five Level IV studies [30, 32, 41, 45, 57]. Risk of bias assessment deemed 13 studies to be low risk [22, 23, 25, 28, 35, 38–41, 43, 48–50], 14 studies were acceptable risk [24, 26, 27, 29, 30, 33, 34, 36, 37, 44–46, 51, 57] and nine studies were high risk studies [31, 32, 42, 47, 52–56]. Studies’ level of evidence and risk of bias were both taken into consideration during quality assessment.

### Study findings

Table 2 summarises the findings from individual studies, of which, 27 studies compared the concurrent diets of study groups (per intervention status or weight outcome) and 16 studies conducted direct tests of association between dietary variable(s) and weight outcome(s). The types of diet being assessed, key findings, and limitations for each included study. Study findings are presented according to the timeframe when dietary intakes were measured post-surgery ≤12-months, between 12 and 24 months, and ≥24 months.

**Table 2:** Study findings on the relationship between weight change and dietary intakes.

Author, Date	Comparison groups or tests of association	Study Findings of Weight- and Diet- Relationships after Bariatric Surgery			Key Findings	Limitations
		≤12 months	Between 12-24 months	≥24 months		
Alvarez et al., 2017 [22]	1. Weight regain (%) at <50^th^ percentile 2. Weight regain (%) at >50^th^ percentile	-	-	Group 1 had lower fat intake (*p* < 0.05). NS differences in energy intake, all other macronutrients, sweet foods, sugar, and alcohol.	Participants with less WR had lower fat intake. NS differences reported for all other measured dietary intakes between comparison groups.	WR is defined by presentation of %WR>50^th^ percentile of the study cohort, which may affect generalizability of findings.
Amundsen et al., 2017 [23]	Comparison 1 1. Excess weight loss ≥50% 2. Excess weight loss <50% Comparison 2 1. Weight regain ≤15% 2. Weight regain >15%	-	-	Comparison 1: Group 1 had higher alcohol intake (*p* < 0.05). NS differences in energy, macronutrients, and all food groups. Comparison 2: NS differences in energy, macronutrients, all food groups, and alcohol intakes.	Participants with greater EWL had higher alcohol intake. NS differences reported for all other measured dietary intakes between comparison groups.	Self-reported weight data.
Bobbioni-Harsch et al., 2002 [24]	Associations with excess weight loss and total weight loss	Positive association between EWL and energy intake (r2 = 0.13, *p* < 0.01). Positive trend between TWL and energy intake (Coefficient 0.01, r2 = 0.56, *p* < 0.07). NS associations between EWL or TWL and macronutrients.	-	-	EWL and TWL are associated with lower energy intake. NS associations reported between EWL and TWL with all other measured dietary intakes.	-
Cadena-Obando et al., 2020 [25]	1. Excess weight loss ≥50% 2. Excess weight loss <50%	NS differences in energy and macronutrients.	-	-	NS differences reported for all measured dietary intakes between comparison groups.	Included only participants with complete medical files.
Chou et al., 2017 [26]	Associations with total weight loss	-	-	Positive trend between TWL and energy (r = 0.313, *p* = 0.052), NS associations with macronutrients.	Greater TWL is associated with higher energy intake. NS associations reported between TWL with all other measured dietary intakes.	Study conducted in the Eastern context and may have less generalizability to the Western context.
	1. Weight regain <25% 2. Weight regain ≥25%			Non-regainers had higher protein and fat (*p* < 0.05), but NS differences in energy or other macronutrient intakes.	Participants with less WR had higher protein and fat intakes. NS differences reported for all other measured dietary intakes between comparison groups.	
Dagan et al., 2017 [27]	1. Excess weight loss ≥60% 2. Excess weight loss <60%	NS differences in proportion of participants consuming ≥60 g/day protein.	-	-	Proportion of subjects having protein intakes of ≥60 g/day did not differ between participants with greater or less EWL.	Self-reported adherence to ≥60 g/day protein intakes. Study was conducted in the Middle-Eastern context and may have less generalizability to the Western context.
da Silva et al., 2010 [28]	Odds of Weight regain ≥10%;	-	-	Inverse association between WR and better diet quality (OR 0.95, 95% CI 0.90–0.99, *p* < 0.05), NS associations with carbohydrate and fat intakes.	Lower odds of WR is associated with a better diet quality, but not with any other measured dietary intakes.	Diet quality measurement tool was designed for use in general population.
	1. Weight regain <10% 2. Weight regain ≥10%			Group 1 had higher fruit (servings/day; *p* < 0.05) intake and better diet quality score (*p* < 0.01). NS differences in energy (kcal/day, kcal/kg ideal weight/day), carbohydrate (%), protein (g/day, g/kg ideal weight/day) and fat (%), vegetables, meats, beans, dairy, grains (*p* = 0.06), sugary and sweets, and fats and oils servings/day.	Participants with less WR had higher fruit intakes and better diet quality score. NS differences reported for all other measured dietary intakes between comparison groups.	
Dodsworth et al., 2012 [29]	Intervention: 6-month prescribed protein-enriched diet (960–1400 kcal/day, 40% carbohydrate, 30% protein, 25% fat). Control: Usual care.	NS differences in BMI, EWL or TWL between groups.	-	-	Protein-enriched diet did not result in greater BMI, EWL, or TWL than having no prescribed diet.	Poor compliance at all timepoints which may affect interpretation of the effect of the prescribed diet.
	Associations with BMI, excess weight loss and total weight loss	NS associations between BMI, EWL, or TWL, with protein intakes.			BMI, EWL or TWL were not associated with protein intakes.	
Faria et al., 2009 [30]	Associations with average monthly weight loss	Moderate inverse associations with total energy intake (kcal/day) (r = 0.373, *p* < 0.01), carbohydrate (g/day) (r = −0.414, *p* < 0.01) and meal glycemic load (r = −0.364, *p* < 0.01). Positive association with protein (%) (r = 0.305, *p* < 0.05) in bivariate analysis (unclear if included in final regression model). NS association with fat (g/day) in final model.		-	Greater average monthly weight loss is associated with lower energy, carbohydrate, meal glycemic load, and higher protein intakes. NS associations reported between greater average monthly weight loss with all other measured dietary intakes.	53% of participants included in the data analysis were less than 12 months post-surgery, which may influence the comparability of findings to other studies where all participants were at least 12 months post-surgery.
Faria et al., 2009 [31]	1. <150 kcal of sweets/snacks between main meals. Highest excess weight loss (%) in cohort (*p* < 0.05 with 3. only). 2. ≥150 kcal of sweets between main meals. 3. ≥150 kcal of snacks between main meals.	-	Group with greatest EWL had lower energy; (*p* < 0.01) and carbohydrate (*p* < 0.01), but NS differences in fat intakes and; lower protein intakes than participants with least EWL(*p* < 0.01).	-	Participants with greater EWL had lower energy, carbohydrate, and protein intakes. NS differences reported for all other measured dietary intakes between comparison groups.	Cohort-specific definition of significant EWL. Differences in EWL reached significance (*p* < 0.05) between highest and lowest EWL groups only.
Faria et al., 2010 [32]	Intervention: 3-month prescribed high protein diet (1400 ± 180 kcal/day, 45% carbohydrate, 35% protein, 20% fat, 3 serves/day dairy). Control: n/a	-	-	On average, participants had significant reduction of BMI (*p* < 0.001) and TBW (*p* < 0.001).	Intervention with prescribed high protein diet lowered the BMI and TBW of participants.	Findings may be confounded by the incentivized physical activity component. No control group. Reduced generalizability, as all participants were highly-motivated with demonstrated high adherence.
Freire et al., 2011 [33]	Associations with excess weight loss	-	-	NS associations between EWL and macronutrient composition (%) or any food groups.	EWL is not associated with macronutrient composition or any food group intakes.	-
	1. Weight regain <2% 2. Weight regain ≥2%			Group 1 had lower energy (kcal/day) (*p* < 0.01), snacks and sweets (*p* < 0.05), and oils and fatty food (*p* < 0.01) servings/week, but NS differences in macronutrient composition, in fruit, vegetable, meats and eggs, beans, dairy, and grain servings/week.	Participants with less WR had lower energy, snacks and sweets, and oils and fatty food intakes. NS differences reported for all other measured dietary intakes between comparison groups.	
Gallé ´ et al., 2020 [34]	Intervention: 12-month exercise, motivational, and nutritional program. Control: Received routine medical examinations only.	Intervention group had greater BMI loss (*p* < 0.01) than control. Intervention group reported increased fruit or vegetables, and reduced sweets servings/day (*p* < 0.05 for all), while controls only reported reduced sweets servings/day (*p* < 0.05). There were NS changes in meat, fish, eggs, dairy, or grain intakes in both groups.	-	-	Lifestyle intervention resulted in greater BMI loss and increased fruit or vegetable, and reduced sweets intakes. NS differences reported for all other measured dietary intakes between comparison groups.	Findings may be confounded by the physical activity component. Intake differences between intervention and control not compared.
Iossa et al., 2020 [35]	Associations with excess weight loss, risk of excess weight loss <50%, risk of weight regain >25%	-	-	Moderate inverse association between EWL with energy (kcal/day; r = −0.54, *p* < 0.05) and fat (g/day; r = −0.35, *p* < 0.05) intakes, but NS associations with carbohydrate (g/day) and protein (g/day) intakes. NS associations between risk of EWL < 50% to energy (kcal/day) and fat (g/day) intakes. Positive association between risk of WR > 25% with energy ( > 1300 kcal/day; HR 4.2, 95% CI 5–16, *p* < 0.05), and fat (g/day; HR 4.2, 95% CI 6–11, *p* < 0.05) intakes.	Greater EWL is associated with lower energy and fat intakes, but not with any other measured dietary intakes. Risk of EWL < 50% is not associated with energy and fat intakes. Higher risk of WR is associated with higher energy and fat intakes.	Energy and fat intakes of Group 1< Group 3< Group 2, but significance was achieved between Groups 1 and 3 only. This may be attributable to the large difference in sample size between groups.
	1. Excess weight loss >50% at first year + <25% regain of excess weight 2. Excess weight loss <50% at first year 3. >25% regain of excess weight loss in absence of surgical complications			NS differences in energy (kcal/day) and fat (g/day) intakes between Groups 1 and 2. Group 1 had lower energy (kcal/day; *p* < 0.05) and fat (g/day; *p* < 0.05) intakes than Group 3.	Participants with greater EWL and less WR had lower energy and fat intakes than those with greater WR only.	
Jassil et al., 2015 [36]	Intervention: 8-week exercise training, and group lifestyle and nutritional behavioural change sessions, at 3–6 months Control: Standard post-surgical care and follow-ups	Intervention group had greater weight and BMI losses between 3–12 months (*p* < 0.05, *p* = 0.05), but not at 12 months. Intervention group reported increased combined fruit and vegetable portions/day, and significantly reduced frequencies of ready meals (*p* < 0.05 for all). There were NS changes in deep-fried foods, crisps, cakes/ biscuits/ chocolate/ sweets, take-away meals, fizzy drinks, fruit juice, and liquid meals.	-	-	Lifestyle intervention resulted in greater weight and BMI losses, increased combined fruit and vegetable, and reduced sweets intake. NS differences reported for all other measured dietary intakes between comparison groups.	Findings may be confounded by the physical activity component. Intake differences between intervention and control not compared.
Kalarchian et al., 2016 [37]	Intervention: 4-month prescribed diet (4 serve/day vegetables, 2–4 serves/day meat, and 1–2 serves/day low glycaemic index grains) with home delivered, portion-controlled meals + usual care Control: Usual care	-	Intervention group had significantly greater weight loss at 16 months (*p* < 0.01) and 18 months (*p* = 0.05). Group allocation had a significant main effect on weight loss from enrolment (F1,31 = 6.79, *p* = 0.01).	-	Intervention with prescribed diet resulted in greater weight loss.	Prescribed diet did not cover all food groups. Interpretation for potential effect of the prescribed diet was not possible. Reduced generalizability, as home-delivered meals were provided as part of study.
Kanerva et al., 2017 [38]	Associations with initial weight loss between men and women, according to changes in intakes from 0–6 months.	At 10 years, greatest IWL were observed from macronutrient composition (%) of carbohydrate > fat (men only; *p* < 0.05), protein > carbohydrate (*p* < 0.05), and protein > fat (*p* < 0.01). Greatest 10-year IWL was achieved by men and women with largest reduction of energy (kcal/day; *p* < 0.001) or carbohydrate (%; *p* < 0.05) intakes, men with largest reduction of fat (%; *p* < 0.001) intakes, women with largest increment of protein (%; *p* < 0.05) and least reduction of alcohol (%; *p* < 0.05) intakes, from 0–6 months.	-	-	Greatest 10-year IWL is associated with lower carbohydrate and fat, and higher protein intakes. Participants with greatest 10-year IWL largely reduced their energy, carbohydrate, and fat (men only) intakes, and increased their protein (women only) intakes, from 0–6 months. Women with greatest 10-year IWL did not reduce their alcohol intakes from 0–6 months.	Absolute differences in IWL (%) between groups were <5% for all measures. These were described by the study as “non-clinically significant”. It was not clear whether adjustment of baseline alcohol intakes were conducted.
Kruseman et al., 2010 [39]	1. Excess weight loss ≥50% 2. Excess weight loss <50%	NS differences in one-year intakes of energy (kcal/day), carbohydrate (%), protein (g/kg/day) and fat (%).	-	Group 1 had lower energy (kcal/day; *p* < 0.05) intakes but NS differences in carbohydrate (%), protein (g/kg/day), and fat (%) intakes.	Participants with greater EWL had lower energy intake at 8-years only. NS differences reported for all other measured dietary intakes between comparison groups.	-
Lim et al., 2020 [40]	1. Excess weight loss ≥50% 2. Excess weight loss <50%;	At 6 months, Group 1 had lower energy (kcal/day; *p* < 0.01), carbohydrate (g/day only; *p* < 0.001) or fat (g/day only; *p* < 0.05), and higher protein (% only; *p* < 0.05) intakes. At 12 months, Group 1 had lower energy (kcal/day; *p* < 0.001), carbohydrate (% and g/day; *p* < 0.001) or fat (% and g/day, *p* < 0.05), and higher protein (% and g/day, *p* < 0.05) intakes.	-	-	Participants with greater EWL had lower energy, carbohydrate and fat, and higher protein intakes.	Study was conducted in the Eastern context and may have less generalizability to the Western context.
	Associations with excess weight loss within participants with excess weight loss ≥50%	There are moderate inverse associations between EWL with energy (kcal/day; r = −0.418, *p* < 0.01) or fat (%; r = −0.273, *p* < 0.001), but NS associations with carbohydrate (%) and protein (%).			In participants with greater EWL, greater EWL was associated with lower energy or fat intakes, but not with carbohydrate or protein intakes.	
	Associations with excess weight loss within participants with excess weight loss <50%	There are moderate inverse associations between EWL with carbohydrate (%; r = −0.3, p < 0.01) or fat (%; r = −0.266, *p* < 0.05), moderate positive associations with protein (%; r = 0.301, *p* < 0.01), and NS associations with energy (kcal/day).			In participants with less EWL, greater EWL was associated with lower carbohydrate or fat intakes, and with higher protein intakes, but not with energy intakes.	
	Associations with odds of excess weight loss ≥50%	There are small inverse associations between the odds of EWL ≥ 50% with carbohydrate (%; OR = 0.99, 95% CI 0.98–0.99, *p* < 0.001) or fat (%; OR = 0.96, 95% CI 0.93–0.98, *p* < 0.001), but NS associations with protein (%).			Greater odds of EWL ≥ 50% are associated with lower carbohydrate and fat intakes, but not with protein intakes.	
	12-month intake cut-offs for reaching excess weight loss ≥50%	Energy of <1523.0 kcal/day (AUC 0.912, 95%CI 0.872-0.953); Carbohydrate of <49% and <172.5 g/day (AUC 0.714, 95%CI 0.637-0.792 / AUC 0.878, 95%CI 0.819-0.937); Protein of >24.5% and >86.5 g/day AUC 0.609, 95%CI 0.523–0.695 / AUC 0.618, 95%CI 0.531–0.705; Fat of <28% and <52.5 g/day (AUC 0.855, 95%CI 0.792-0.917 / AUC 0.781, 95%CI 0.709-0.853).			At 12-months, intake cut-offs for participants with EWL > 50% were <1523.0 kcal, 49% carbohydrates, >24.5% protein and <28% fats per day.	
Lindroos et al., 1996 [41]	Associations with weight change	-	Positive association between weight change (kg) with polysaccharides (%) (t = 2.05, *p* < 0.05), protein (%) (t = 2.94, *p* < 0.01), or prepared meals (%) (t = 4.59, *p* < 0.001). Inverse association between weight change (kg) with mono/disaccharides (%) (t = −3.16, *p* < 0.01), fat (%) (t = −2.11, *p* < 0.05) or sweet foods (%) (t = −3.61, *p* < 0.001). NS associations with energy (kcal/day), sandwiches (%), and alcohol (%).	-	Positive weight change (weight gain) is associated with higher intakes of polysaccharides, protein or prepared meals. Negative weight change (weight loss) is associated with higher intakes of mono/disaccharides, fat, and sweet foods. NS associations reported between weight change with all other measured dietary intakes.	Direct comparison for carbohydrate intakes with other studies was not possible due to intakes being divided into mono/disaccharides and polysaccharides.
	Total weight loss between participants with highest vs lowest quartiles of intakes		Greater TWL (*p* < 0.05) achieved from higher intakes of mono/disaccharides ( > 142 vs <72 g/day), fats ( > 96 vs <51 g/day), sweet foods ( > 2.83 vs <1.09MJ/day), and lower intakes of polysaccharides ( < 79 vs >132 g/day), protein ( < 56 vs >92 g/day), and prepared meals ( < 1.06 vs >2.08MJ/day). Alcohol (0 g/day vs >6.7 g/day) and sandwiches ( < 0.9MJ/day vs >2.31MJ/day) did not result in differences in TWL (kg).		Greater weight losses are achieved by higher intakes of mono/disaccharides, fats or sweet foods, and lower intakes of polysaccharides, proteins and prepared meals. NS differences reported for all other measured dietary intakes between comparison groups.	
Masood et al., 2019 [42]	1. Weight regain <15% 2. Weight regain ≥15%	-	Higher proportion of weight loss maintainers consumed fat (*p* < 0.01), fruit (*p* < 0.001), and vegetable (*p* < 0.001) intakes of 3–5 exchanges/day. NS differences in the proportion of participants eating carbohydrate of 1–5 exchanges/day, or ready-to-eat foods and fast foods of 0–1 times/week.	-	Participants who experienced less WR consumed fats, fruits, and vegetable intakes of 3–5 exchanges/day. NS differences reported for all other measured dietary intakes between comparison groups.	Portion size of each exchange not specified. Study was conducted in the Middle-Eastern context and may have less generalizability to the Western context.
Moizé, V. et al., 2013 [43]	Associations with excess weight loss	-	-	Inverse association with energy (kcal/day; r-value NR, *p* < 0.01), but NS association with macronutrient composition (%).	Greater EWL is associated with lower energy intake. NS associations reported between EWL with all other measured dietary intakes.	The strength of association was not reported.
Novais et al., 2012 [44]	1. Excess weight loss ≥75% 2. Excess weight loss 50-75% 3. Excess weight loss <50%	-	-	NS differences in energy (kcal/day) and macronutrient composition (%).	Participants with greater EWL did not have different dietary intakes than those with less EWL.	-
Ortega et al., 2012 [45]	Associations with BMI loss.	-	-	Inverse associations with energy (kcal/day; B = −0.003, *p* < 0.01) intake. NS associations with macronutrient composition (%).	Greater BMI loss is associated with lower energy intakes. NS associations reported between BMI loss with all other measured dietary intakes.	Self-reported weight data.
Palacio et al., 2020 [46]	Associations with odds of weight regain ≥15%	-	-	Positive associations with energy (kcal/day; OR 1.3, 95% CI 1.1–1.9, *p* < 0.05) but NS associations with carbohydrate (% and g/day) intakes.	Greater odds of WR is associated with higher energy intake. NS associations reported between odds of WR with all other measured dietary intakes.	Weight data measured at 2 years but compared to diet data collected at 7-years.
	1. Weight regain <15% 2. Weight regain ≥15%			Group 1 had lower 7-year energy (kcal/day; *p* < 0.001) and carbohydrate (% −*p* < 0.01, and g/day −*p* < 0.001) intakes.	Participants with less WR had lower energy and carbohydrate intakes.	
Papalazarou et al., 2010 [47]	Intervention: 3-years person-centred lifestyle behaviour change program + usual care Control: Usual care	Intervention group had greater EWL (*p* < 0.05) and lower TBW (*p* < 0.05), but NS differences in fruit, vegetable, and sweets servings/day than control. Group allocation remained the only significant factor in weight losses (*p*-value NR).	-	Intervention group had greater EWL and lower TBW, higher fruit and vegetable, and lower sweet servings/day (*p* < 0.05 for all) than control. Group allocation remained the only significant factor in weight losses (*p*-value NR).	Lifestyle intervention resulted in greater EWL, lower TBW, higher fruit and vegetable intakes, and lower sweet intakes.	Intervention group had higher physical activity levels than control at three years (*p* < 0.05). This may have confounded results at three-years.
Pinto et al., 2019 [48]	Associations with risk of obesity remission (BMI < 30)	Positive association with 0–3 months changes in protein (%; HR = 1.06, 95% CI 1.01–1.12, *p* < 0.05) intakes, but NS association with 0–3 months changes in carbohydrate (%) and fat (%).	-	-	Greater risk of obesity remission is associated with higher protein intakes. NS associations reported between risk of obesity remission with all other measured dietary intakes.	-
Raftopoulos et al., 2011 [49]	Associations with BMI change and excess weight loss	Positive association with protein (g/kg/day) intakes (BMI change: B = 2.46, 95%CI 1.32–3.69, R2 = 0.041, *p* < 0.001; EWL: B = 8.28, 95%CI 3.65–12.92, R2 = 0.054, *p* < 0.01). Positive association with consistent compliance to ≥1 g/kg/day protein intakes (BMI change: F = 5.097, *p* < 0.01; EWL: F = 4.415, *p* < 0.01).	-	-	Greater BMI change and EWL are associated with higher protein intakes and more consistent compliance to ≥1 g/kg/day protein.	12-month data was only available for 27% of participants with incomplete follow-up.
Reid et al., 2016 [50]	1. Total weight loss ≥38% 2. Total weight loss ≤30%	-	-	Group 1 had lower carbohydrate (g/day; F1,23 = 5.065, *p* < 0.05) and alcohol (g/day; F1,23 = 4.836, *p* < 0.05) intakes. NS differences in energy (kcal/day), carbohydrate (%), protein (%, g/day, % of participants with intake of ≥60 g/day), and fat (% and g/day) intakes.	Participants with greater TWL had lower carbohydrate and alcohol intakes. NS differences reported for all other measured dietary intakes between comparison groups.	Diet data were measured one-year after collection of weight data.
Ruiz-Lozano et al., 2016 [51]	1. Excess weight loss ≥50% at nadir and until last follow-up 2. Excess weight loss <50% at nadir and up to last follow-up 3. Excess weight loss ≥50% at nadir but <50% at last follow-up		NS difference in energy and macronutrient composition (% and g/day).	NS difference in energy and macronutrient composition (% and g/day).	Participants with greater EWL at nadir and at last-follow-up did not have different dietary intakes to those with less EWL.	No assessment of exact amount of WR. It is possible that significant amounts of WR were present in achievers despite maintaining >50%EWL at last follow-up.
Sarwer et al., 2012 [52]	Intervention: 4-month dietary counselling sessions + usual care Control: Usual care	NS differences between groups in IWL, energy (kcal/day), macronutrient composition (%) and sweet (%).		-	Intervention did not result in greater or less EWL, or higher or lower dietary intakes.	Did not result in any significant changes to enable determination of presence or absence of associations.
Schiavo et al., 2016 [53]	Intervention: 12-month prescribed protein-enriched diet (1200 kcal/day, 37.3% carbohydrate, 47.7% protein, 15% fat) Control: 12-month prescribed normal-protein diet (1200 kcal/day, 61.7% carbohydrate, 23.3% protein, 15% fat)	NS differences in TBW between groups.	-	-	Protein-enriched diet did not result in lower TBW than normal-protein diet.	All participants were of male gender. Findings may be less comparable to all other included studies which had higher proportion of female participants.
Schiavo et al., 2018 [54]	1. Prescribed low-purine diet (890 kcal/day, 55% carbohydrate, 20% protein, 25% fat, emphasis on low-purine foods) 2. Prescribed normal-purine diet (890 kcal/day, 55% carbohydrate, 20% protein, 25% fat)	NS differences between groups in BMI and TBW.	-	-	Low-purine diet did not result in lower BMI or TBW than normal-purine diet.	Adherence assessment was based on prescribed energy and macronutrient, instead of purine intakes.
Taus et al., 2017 [55]	Intervention: 2-month prescribed ketogenic diet (800 kcal/day, 20% carbohydrate, 40% protein, 40% fat) Control: Prescribed usual care diet 800 kcal/day, 52% carbohydrate, 25% protein, 23% fat + Band calibration (Average 8cc)	-	Intervention had lower BMI, greater EWL, and lower TBW than control (*p*-values NR for all measures).	-	Ketogenic diet resulted in lower BMI and TBW and greater EWL than usual care diet.	At baseline, intervention group had lower BMI and TBW and greater EWL, than control. However, significance in weight differences between- and within- group, at baseline and at after intervention, were not described.
Wardé-Kamar et al., 2004 [56]	Associations with excess weight loss	-	-	Inverse associations with energy (kcal/day) (p-value NR). Predictors of 47% EWL were: Age, excess weight, pre-surgery weight, and energy (kcal/day) and fat (%) intakes (*p* < 0.001).	Greater EWL was associated with lower energy intakes. Energy and fat intakes were some of the predictors of 47% of EWL post-surgery. NS associations reported between EWL with all other measured dietary intakes.	Self-reported weight data. Interpretation of strength and direction of association (fat only) were not possible due to no reporting of respective statistics.
	1. Excess weight loss ≥50% 2. Excess weight loss <50%			NS differences in energy (kcal/day) and macronutrient composition (%).	Participants with greater EWL did not have different dietary intakes to those with less EWL.	
Yanos et al., 2015 [57]	Associations with total weight loss at nadir	-	-	NS associations between TWL at nadir, with having ≥60–80 g/day protein, ≥5 serves/day of fruit or vegetables, or avoiding sweets.	TWL at nadir is not associated with any measured intake variables.	Self-reported weight data.
	Associations with risk of weight regain ≥20%			Avoidance of sweets was an independent predictor for risk of WR ≥ 20% (r2 = 0.22, *p* < 0.01; bivariate analysis: r = −0.28, *p* < 0.01). NS associations with having ≥60–80 g/day protein, ≥5 serves/day of fruit, or ≥5 serves/day of vegetables.	Lower risk of WR ≥ 20% is associated with avoidance of sweets. NS associations reported between risk of WR ≥ 20% with all other measured dietary intakes.	

*AUC* Area under curve, *BM*I Body mass index, *CI* Confidence interval, *EWL* Excess weight loss, *IWL* Initial weight loss, *n/a* Not applicable, *NR* Not reported, *NS* Non-significant, *OR* Odds ratio, *TBW* Total body weight, *TWL* Total weight loss, *WR* Weight regain.

### Diet ≤12-months post-surgery and weight loss

Sixteen studies described diets up to 12-month and observed weight losses up to ten-years [24, 25, 27, 29, 30, 34, 36, 38–40, 47–49, 52–54]. Regarding carbohydrate, significant inverse associations with weight losses were supported by three observational cohort studies (total *N* = 1888, up to 10-years follow-up) [30, 38, 40]. However, one RCT (prescribed protein enriched, low-carbohydrate diet vs prescribed normal protein and carbohydrate diet, with high reported adherence) and four observational cohort studies showed no significant associations (total *N* = 368, up to 8-years follow-up) [24, 25, 39, 48, 53]. Regarding protein, significant positive associations with weight loss was supported by five observational cohort studies (total *N* = 2232, up to 10-years follow-up) [30, 38, 40, 48, 49]. On the contrary, one RCT (prescribed protein enriched, low-carbohydrate diet vs prescribed normal protein- and carbohydrate diet, with high reported adherence), one pseudo-RCT (prescribed protein-enriched diet vs no prescribed diets, with poor reported adherence), and four observational cohort studies did not report significant associations with weight loss (total *N* = 444, up to 8-years follow-up) [24, 25, 27, 29, 39, 53]. Regarding fat, significant inverse associations with weight losses were supported by two observational cohort studies (total *N* = 1799, up to 10-years follow-up) [38, 40]. However, five observational cohort studies did not report any significant associations (total *N* = 400, up to 8-years follow-up). A single RCT that involved a lifestyle intervention did not result in any significant between-group differences in macronutrient intakes nor weight changes[52].

Food pattern, specifically fruit and vegetable intakes, were reported in three studies (one RCT, two non-randomized controlled trials) [34, 36, 47]. All studies reported significantly greater weight losses in intervention groups compared with controls after lifestyle interventions [34, 36, 47]. However, no significant differences in fruit and vegetable intakes were noted between intervention and control groups within the single RCT [47]. Although between-group intake differences were not measured within the two non-randomized controlled trials, both studies reported significant increases in fruit and vegetable intakes in their intervention groups when compared to pre-intervention intakes [34, 36]. As both studies included a physical activity component, it was not possible to attribute outcomes to diet alone [34, 36]. There was inadequate information provided on other food patterns including dairy, meat and grains, as they were not measured in the single RCT [47], whereas the two non-randomized controlled trials reported no significant changes in their intakes compared to pre-intervention [34, 36]. Lastly, a single cohort study observed the effect of varying purine contents of the diet (exact food group intakes not reported), which did not show any significant association with post-surgery weight [54].

### Diet ≤12-months post-surgery and weight recurrence

No studies examined weight recurrence during this period, presumably due to the short timeframe to enable observation of weight recurrence.

### Diet between 12- and 24-months post-surgery and weight loss

Six studies described diets between 12- and 24-months post-surgery and observed weight losses up to five years post-surgery [31, 37, 41, 51, 52, 55]. Diet was also described and compared with weight recurrence at two years post-surgery by a single cohort study [42]. Regarding carbohydrate, significant inverse associations with weight losses was supported by one observational cohort study (total *N* = 75, with up to 2-years follow-up), whereas a lack of association was suggested by one non-randomized controlled trial and one observational cohort study (total *N* = 290, with up to 5-years follow-up). Additionally, Lindroos et al. assessed the potential impact of different types of carbohydrate intakes and reported greater weight loss for participants with higher intakes of mono- or di-saccharides and lower intakes of polysaccharides, though the potential impacts of total carbohydrate intake was not assessed [41]. Findings for protein included one observational cohort study (*N* = 75, with up to 2-years follow-up) suggesting positive associations [31], one observational cohort study (*N* = 375, with up to 2-years follow-up) suggesting inverse associations [41], and two studies (one non-randomized controlled trial and one observational cohort) suggesting no significant associations with weight loss (total *N* = 290, with up to 5-years follow-up) [51, 55]. Regarding fat, only one observational cohort study (*N* = 375, up to two-years) [41] supported positive associations with weight loss whereas three studies (one non-randomized controlled trial, two observational cohorts) reported having no significant associations (total *N* = 365, up to 5-years) [31, 51, 55]. A single RCT that involved a lifestyle intervention did not result in any significant between-group differences in macronutrient intakes nor weight outcomes [52]. The relationship between post-surgery weight loss and food pattern was assessed by a single RCT [37], providing monthly home delivered meals with a personalized menu plan of 4 serves vegetables, 2–4 serves meat, and 1–2 serves grains per day. The intervention resulted in greater weight loss than a control group with no delivered meals or prescribed diet and may indicate some benefits for similar types of intervention or prescribed menu plans [37].

### Diet between 12- and 24-months post-surgery and weight recurrence

Macronutrient composition, food pattern, and weight recurrence at up to 18-months post-surgery were described by a cohort study [42]. The findings of this study favored a diet with daily intakes of 3–5 fat, fruit, and vegetable exchanges for less weight recurrence, though the portion size of each food exchange was not reported [42].

### Diet at ≥24-months post-surgery and weight loss

Fourteen studies described dietary intakes after 24-months post-surgery and weight loss up to 12 years post-surgery [23, 26, 32, 33, 35, 39, 43–45, 47, 50, 51, 56, 57]. The absence of association was supported by 11 out of 11 observational cohort studies regarding carbohydrate and fat intakes (Total *N* = 1305, with up to 12-years follow-up) [23, 26, 33, 35, 39, 43–45, 50, 51, 56], and by 12 out of 12 observational cohort studies for protein intake (Total *N* = 1402, with up to 12-years follow-up) [23, 26, 33, 35, 39, 43–45, 50, 51, 56, 57]. A pre-/post-interventional study of individuals who had experienced weight recurrence at 3 years post-surgery reported significant weight loss from a three-month diet with 45% carbohydrate, 35% protein, and 20% fat [32]. Despite high adherence, the researchers did not compare to pre-intervention intakes, and an incentivized physical activity component made attribution of results to diet alone impossible [32]. Regarding food pattern, the absence of association between weight loss and any core food group, except fruit and vegetables, was supported by 2 out of 2 observational cohort studies (total *N* = 147, with up to 5 years follow-up) [23, 33]. A positive association between weight loss and fruit and vegetables was suggested by one RCT, where a lifestyle intervention resulted in significantly greater weight loss and concurrent significantly higher fruit and vegetable intakes in the intervention group compared to controls (*N* = 30, with up to 3 years follow-up) [47]. However, the component of physical activity prevented the outcomes being attributed to diet alone [47]. Despite this, three cohort studies reported no associations between fruit or vegetable intakes with weight loss up to 9-years (total *N* = 244, with up to 9-years follow-up) [23, 33, 57].

### Diet at ≥24-months post-surgery and weight recurrence

Eight observational cohort studies described dietary intakes after 24-months and observed weight recurrence up to 9 years [22, 23, 26, 28, 33, 35, 46, 57]. For macronutrient intake composition, the lack of association between weight recurrence with carbohydrate and protein intakes was largely consistent across studies: Carbohydrate (6 out of 6 cohort studies, total *N* = 410, with up to five-years follow-up [22, 23, 26, 28, 33, 46]); Protein (5 [22, 23, 28, 33, 57] out of 6 cohort studies [22, 23, 26, 28, 33, 57], total *N* = 366 out of 403, with up to 9 years follow-up). The findings regarding fat intakes were mixed, though the absence of association was supported by 4 [22, 23, 28, 33] out of 6 cohort studies [22, 23, 26, 28, 33, 35] (total *N* = 269 out of 392, with up to 7-years follow-up [22, 23, 28, 33]). Differences in the definition of significant weight recurrence across studies (i.e. >2% to >25% recurrence of weight [22, 23, 26, 28, 33, 35]) may partially account for the mixed results. Notably, in the cohort study with the longest follow-up (7-years) and strictest criteria for Group 1 (>50% excess weight loss at first year and maintaining <25% weight recurrence until 7-years), higher fat intakes, in addition to total energy intake, were linked to a 4-fold increased risk of weight recurrence within a cox hazard regression model. Group 1 participants also reported significantly lower intakes of fat than Group 2 participants (>25% weight recurrence at 7-years).

Regarding food patterns, a lack of association between weight recurrence and any core food group was reported by 4 out of 4 observational cohort studies (total *N* = 326, with total follow-up of 8.9 years) [23, 28, 33, 47], except for fruit, where 1 out of the 4 studies was suggestive of an inverse association between fruit intakes and weight recurrence [28]. Diet quality score, measured using the Brazilian version of the Healthy Eating Index, was assessed by one cohort study that showed a weak inverse association with the odds of weight recurrence with a higher diet quality score (achieved by having a balanced daily servings of grains, vegetables, fruits, beans, meats, dairy products, fats and oils, sugar and sweets, restricted intakes of saturated fats and cholesterol, and having a high variety of foods within the diet) [28].

### Grading of evidence and recommendations

Tables 3 to 6 provide a summary of the results assessed using the NHMRC body of evidence framework [20].

**Table 3:** Grading of Evidence for associations between dietary intakes ( ≤ 12 months) and weight loss (up to ten-years) (15 studies)*.

	Macronutrient Composition			Food Pattern
	Carbohydrate	Protein	Fat	Fruit and Vegetables
Study Findings	Inverse Association (3 studies, total *N* = 1888, up to 10-years) [30–38, 40] Level II: 1 LR study [38] (*N* = 1610) Level III: 1 LR study [40] (*N* = 189) Level IV: 1 AR study [30] (*N* = 89) No Association (5 studies, total *N* = 368, up to 8-years) [24, 25, 39, 48, 53] Level II: 1 LR study [48] and 2 AR studies [24, 53] (*N* = 161) Level III: 2 LR studies [25, 39] (*N* = 207)	Positive Association (5 studies, total *N* = 2232, up to 10-years) [30, 38, 40, 48, 49] Level II: 3 LR studies [38, 48, 49] (*N* = 1954) Level III: 1 LR study [40] (*N* = 189) Level IV: 1 AR study [30] (*N* = 89) No Association (6 studies, total *N* = 444, up to 8-years) [24, 25, 27, 29, 39, 53] Level II: 2 AR studies [24, 53] (*N* = 110) Level III: 2 LR studies [25, 39] and 2 AR studies [27] (*N* = 334)	Inverse Association (2 studies, total *N* = 1799, up to 10-years) [38, 40] Level II: 1 LR study [38] (*N* = 1610) Level III: 1 LR study [40] (*N* = 189) No Association (5 studies, total *N* = 400, up to 8-years) [24, 25, 30, 39, 48] Level II: 2 AR studies [24, 48] (*N* = 101) Level III: 2 LR studies [25, 39] (*N* = 210) Level IV: 1 AR study [30] (*N* = 89)	Positive Association (2 studies, total *N* = 94, up to 1.5 years) [34, 36] Level III: 2 AR studies [34, 36] (*N* = 94)
GRADING OF EVIDENCE				
Evidence Statement	An evidence statement could not be made due to inconsistent evidence.			
Evidence Base	C – Satisfactory. Level II to IV (majority being cohort) studies with low to acceptable risk of bias.			C – Satisfactory. Level III (intervention) studies with acceptable risk of bias.
Consistencies	D – Poor. Study findings highly inconsistent. Multiple study designs with varied risk of bias, outcome measures and duration of follow-up.			A – Excellent. All study findings were consistent. Similar study design, risk of bias, outcome measures and duration of follow-up.
Clinical Impact	D – Poor. Inconsistent study findings and design have affected ability to apply to practice. Non-diet moderators to weight change not always addressed.			C – Slight. Though consistent findings, co-variables from study design have prevented attribution of effect to diet intakes alone.
Generalizability	B – Good. All studies in adults at least one-year post-bariatric surgery in an outpatient setting.			C – Slight All studies in adults at least one-year post-bariatric surgery in an outpatient setting. Inclusion of only motivated individuals in one of the study’s interventions.
Applicability	B – Good. Most studies were conducted with population from the Western context like the Australian bariatric context.			
RECOMMENDATION				GRADE OF RECOMMENDTION
No recommendations could be drawn. More well designed RCTs or prospective cohort studies may help with clarifying whether and what associations may be present between weight and diet within ≤12 months post-bariatric surgery.				-

*Using NHMRC body of evidence framework [20]. *AR* Acceptable risk of bias, *HR* High risk of bias, *LR* Low risk of bias.

**Table 4:** Grading of Evidence for associations between dietary intakes (Between 12 and 24months) and weight loss (up to five-years – 4 studies) or weight recurrence (up to 18-months – one study)*.

Grading of Evidence for associations between dietary intakes (Between 12 and 24months) and weight loss (up to five-years – 4 studies)				
	Macronutrient Composition			Food Pattern
	Carbohydrate	Protein	Fat	Nil studies
Study Findings	Inverse Association (1 study, total *N* = 75, up to 2-years) Level III: 1 HR study [31] (*N* = 75) No Association (2 studies, total *N* = 290, up to 5-years) Level III: 1 AR study [51], 1 HR study [55] (*N* = 290)	Positive Association (1 study, total *N* = 75, up to 2-years) Level III: 1 HR study [31] (*N* = 75) Inverse Association (1 study, total *N* = 375, up to 2-years) [41] Level IV: 1 LR study [41] (*N* = 375) No Association (2 studies, total *N* = 290, up to 5-years) Level III: 1 AR study [51], 1 HR study [55] (*N* = 290)	Positive Association (1 study, total *N* = 375, up to 2-years) [41] Level IV: 1 LR study [41] (*N* = 375) No Association (3 studies, total *N* = 365, up to 5-years) Level III: 1 AR study [51], 2 HR studies [31, 55] (*N* = 365)	
NHMRC Body of Evidence Framework				
Evidence Statement	An evidence statement could not be made due to inconsistent evidence.			-
Evidence Base	D - Poor. Level III to IV (cohort) studies with low to high risk of bias.			-
Consistencies	D – Poor. Study findings highly inconsistent. Multiple study designs with varied risk of bias, outcome measures and duration of follow-up.			
Clinical Impact	D – Poor. Inconsistent study findings and design have affected ability to apply to practice. Non-diet moderators to weight change not always addressed.			
Generalizability	B – Good. All studies in adults at least one-year post-bariatric surgery in an outpatient setting.			
Applicability	B – Good. Most studies were conducted with population from the Western context like the Australian bariatric context.			
RECOMMENDATION				GRADE OF RECOMMENDTION
No recommendations could be drawn. More well designed RCTs or prospective cohort studies may help with clarifying whether and what associations may be present between weight and diet within 12 and 24 months post-bariatric surgery.				-
Grading of Evidence for associations between dietary intakes (Between 12 and 24months) and weight recurrence (up to 18-months – one study)				
	Macronutrient Composition			Food Pattern
	Carbohydrate	Protein	Fat	Fruit and Vegetables
Study Findings	No Association with intakes of 1-5 exchanges a day (1 study, total *N* = 50, up to 18-months) Level III: 1 HR study [42] (*N* = 50)	Nil studies	Inverse Association with intakes of 3–5 exchanges a day (1 study, total *N* = 50, up to 18-months) Level III: 1 HR study [42] (*N* = 50)	Inverse Association with intakes of 3–5 exchanges a day (1 study, total *N* = 50, up to 18-months) Level III: 1 HR study [42] (*N* = 50)
NHMRC Body of Evidence Framework				
Evidence Statement	An evidence statement could not be made due to inadequate information from study (unknown portion size for each exchange; unknown outcomes of participants with intakes lower or higher than the specified range of food exchanges) to determine the associations between weight recurrence and dietary intakes.			
Evidence Base	D – Poor. Level III cohort study with high risk of bias.			
Consistencies	N/A Evidence derived from single cohort study.			
Clinical Impact	D – Poor. Weak evidence base have affected ability to apply to practice. No description of the portion size of a single exchange, which have prevented the determination of the potential associations between weight and dietary intakes.			
Generalizability	D – Poor. All studies in adults at least one-year post-bariatric surgery in an outpatient setting. Insufficient dietary information to allow reproducibility.			
Applicability	C – Limited. Study was conducted in the Middle Eastern context which may warrant some caveats to adapt to the Australian bariatric context.			
RECOMMENDATION				GRADE OF RECOMMENDTION
No recommendations could be drawn. More well designed RCTs or prospective cohort studies may help with clarifying whether and what associations may be present between weight and diet within 12 and 24 months post-bariatric surgery.				-

^*^Using NHMRC body of evidence framework [20]. *AR* Acceptable risk of bias, *HR* High risk of bias, *LR* Low risk of bias.

**Table 5:** Grading of Evidence for associations between dietary intakes ( ≥ 24 months and beyond) and weight loss (up to twelve-years) (13 studies)^*^.

	Macronutrient Composition			Food Pattern	
	Carbohydrate	Protein	Fat	Fruit and Vegetables	All non-fruit or vegetable core food groups
Study Findings	No Association (11 studies, total *N* = 1305, up to 12-years) [23, 26, 33, 35, 39, 43–45, 50, 51, 56] Level II: 1 LR study [43] (*N* = 355) Level III: 4 LR studies [23, 35, 39, 50], 4 AR studies [26, 33, 44, 51], 1 HR study [56] (*N* = 843) Level IV: 1 AR study [45] (*N* = 107)	No Association (12 studies, total *N* = 1402, up to 12-years) [23, 26, 33, 35, 39, 43–45, 50, 51, 56, 57] Level II: 1 LR study [43] (*N* = 355) Level III: 4 LR studies [23, 35, 39, 50], 4 AR studies [26, 33, 44, 51], 1 HR study [56] (*N* = 843) Level IV: 2 AR studies [45, 57] (*N* = 204)	No Association (11 studies, total *N* = 1305, up to 12-years) [23, 26, 33, 35, 39, 43–45, 50, 51, 56] Level II: 1 LR study [43] (*N* = 355) Level III: 4 LR studies [23, 35, 39, 50], 4 AR studies [26, 33, 44, 51], 1 HR study [56] (*N* = 843) Level IV: 1 AR study [45] (*N* = 107)	Positive Association (1 study, total *N* = 30, up to 3-years) Level II: 1 HR study [47] No Association (3 studies, total *N* = 244, up to 9-years) Level III: 1 LR study [23], 1 AR study [33] (*N* = 147) Level IV: 1 AR study [57] (*N* = 97)	No Association (2 studies, total *N* = 147, up to 5-years) Level III: 1 LR study [23], 1 AR study [33] (*N* = 147)
GRADING OF EVIDENCE					
Evidence Statement	Weight loss is not associated with macronutrient composition at >24 months post-bariatric surgery.			Weight loss is not associated with core food group patterns at >24 months post-bariatric surgery.	
Evidence Base	B – Good. >2 Level III (cohort) studies with low risk of bias. Study findings are derived from cohort studies only.			C – Satisfactory. 1-2 Level III (cohort) studies with low risk of bias. Though evidence base included one interventional study, it was one that was of an increased risk of bias with small sample size.	
Consistencies	A – Excellent 11 out of 11 studies demonstrated no associations between weight loss and carbohydrate intake.	A – Excellent 12 out of 12 studies demonstrated no associations between weight loss and protein intake.	A – Excellent 11 out of 11 studies demonstrated no associations between weight loss and fat intake.	B – Good. 3 out of 4 studies demonstrated no associations between weight loss and fruit/vegetable intakes. Co-variables from study design have prevented attribution of effect to diet intakes alone in the single study that demonstrated an association.	A – Excellent 2 out of 2 studies demonstrated no associations between weight loss and non-fruit/vegetable intakes.
Clinical Impact	B – Good. Study findings highly consistent regardless of participant characteristics, study design, risk of bias, outcome measures or duration of follow-up.				
Generalizability	B – Good. All studies in adults at least one-year post-bariatric surgery in an outpatient setting.				
Applicability	B – Good. Most studies were conducted with population from the Western context like the Australian bariatric context.				
RECOMMENDATION					GRADE OF RECOMMENDTION
Individualized diets with flexibility on macronutrient and food group composition can be recommended at two years or more post-bariatric surgery, as no composition or patterns showed associations with weight loss.					B Body of evidence can be trusted to guide practice in most situations.

^*^Using NHMRC body of evidence framework [20]. *AR* Acceptable risk of bias, *HR* High risk of bias, *LR* Low risk of bias.

**Table 6:** Grading of Evidence for associations between dietary intakes ( ≥ 24 months and beyond) and weight recurrence (up to 9-years) (6 studies)*.

	Macronutrient Composition				Food Pattern	
	Carbohydrate	Protein	Fat	Fruit	All non-fruit core food groups	Diet Quality
Study Findings	No Association (6 studies, total *N* = 410, up to 5-years) [22, 23, 26, 28, 33, 46] Level III: 3 LR studies [22, 23, 28], 3 AR studies [26, 33, 46] (*N* = 410)	Inverse Association (1 study, total *N* = 37, up to 5-years) Level III: 1 AR study [26] (*N* = 37) No Association (5 studies, total *N* = 366, up to 9-years) [22, 23, 28, 33, 57] Level III: 3 LR studies [22, 23, 28], 1 AR study [33] (*N* = 269) Level IV: 1 AR study [57] (*N* = 97)	Positive Association (1 study, total *N* = 37, up to 5-years) Level III: 1 AR study [26] (N = 37) Inverse Association (1 study, total *N* = 86, up to 7-years) Level III: 1 LR study [35] (*N* = 86) No Association (4 studies, total *N* = 269, up to 5-years) [22, 23, 28, 33] Level III: 3 LR studies [22, 23, 28], 1 AR study [33] (*N* = 269)	Inverse Association (1 study, total *N* = 80, up to 3-years) [28] Level III: 1 LR study [28] (N = 80) No Association (3 studies, total *N* = 246, up to 9-years) [23, 33, 57] Level III: 1 LR study [23], 1 AR study [33] (*N* = 149) Level IV: 1 AR study [57] (*N* = 97)	No Association (4 studies, total *N* = 326, up to 9-years) [23, 28, 33, 57] Level III: 2 LR studies [23, 28], 1 AR study [33] (*N* = 229) Level IV: 1 AR study [57] (*N* = 97)	Inverse Association (1 study, total *N* = 80, up to 3-years) [28] Level III: 1 LR study [28] (*N* = 80)
GRADING OF EVIDENCE						
Evidence Statement	Weight recurrence is not associated with carbohydrate or protein intakes at >24 months post-bariatric surgery.		An evidence statement could not be made due to inconsistent evidence.	Weight recurrence is not associated with core food group pattern at >24 months post-bariatric surgery.		Weight recurrence is inversely associated with a higher quality diet at >24months post-bariatric surgery.
Evidence Base	B – Good. >2 Level III (cohort) studies with low risk of bias. Study findings are derived from cohort studies only.			C – Satisfactory. 1-2 Level III (cohort) studies with low risk of bias. Study findings are derived from cohort studies only.		C – Satisfactory. 1 Level III (cohort) study with low risk of bias. Study finding derived from single cohort study only.
Consistencies	A – Excellent 6 out of 6 studies demonstrated no associations between weight recurrence and carbohydrate intake.	B – Good. 5 out of 6 studies demonstrated no associations between weight recurrence and protein intake.	D – Poor. Study findings highly inconsistent. ultiple study designs with varied risk of bias.	B – Good. 3 out of 4 studies demonstrated no associations between weight recurrence and fruit intake.	A – Excellent 4 out of 4 studies demonstrated no associations between weight recurrence and non-fruit food groups.	N/A Evidence derived from single study only.
Clinical Impact	B – Good. Study findings highly consistent regardless of participant characteristics, study design, risk of bias, outcome measures or duration of follow-up.		D – Poor. Inconsistent study findings and design have affected ability to apply to practice.	B – Good. Study findings highly consistent regardless of participant characteristics, study design, risk of bias, outcome measures or duration of follow-up.		D – Poor. Evidence was derived from single cohort study only, which have affected ability to apply to practice.
Generalizability	B – Good. All studies in adults at least one-year post-bariatric surgery in an outpatient setting.					
Applicability	B – Good. Most studies were conducted with population from the Western context like the Australian bariatric context.					
RECOMMENDATION 1						GRADE OF RECOMMENDTION
Individualized diets with flexibility on macronutrient and food group composition can be recommended at two years or more post-bariatric surgery, as no composition or patterns showed associations with weight regain.						C Body of evidence provides some support for recommendation(s) but care should be taken in its application.
RECOMMENDATION 2						GRADE OF RECOMMENDTION
Weight recurrence may be reduced with a better-quality diet, but more well designed RCTs or prospective cohort studies are required to strengthen this evidence base.						D Body of evidence is weak, and recommendation must be applied with caution.

*Using NHMRC body of evidence framework [20]. *AR* Acceptable risk of bias, *HR* High risk of bias, *LR* Low risk of bias.

The body of evidence for diet ≤12 months post-surgery and weight loss is summarized in Table 3. Overall, the body of evidence was inconsistent regarding the presence or absence of any positive or negative associations between weight loss and macronutrient composition. While the findings for food group patterns appeared to be consistent, co-variables related to study design prevented the effect from being attributed to dietary intake alone. Therefore, no recommendations could be drawn.

The body of evidence for diet between 12- and 24-months post-surgery and weight loss and weight recurrence is summarized in Table 4. The body of evidence was inconsistent regarding the presence or absence of any associations between weight loss and diet, and there was inadequate information to draw conclusions on whether, and in what way, dietary intakes were associated with weight recurrence. Therefore, no recommendations could be drawn.

The body of evidence for diet ≥24 months post-surgery and weight loss is summarized in Table 5. The overall body of evidence was consistent for the absence of any significant associations between weight loss and all macronutrient intakes and food patterns. This body of evidence contributed to the recommendation that long-term diets post-bariatric surgery can be individualized with flexibility as to macronutrient and food pattern composition (Grade B –the body of evidence can be trusted to guide practice in most situations).

The body of evidence for diet ≥24 months post-surgery and weight recurrence is summarized in Table 6. The body of evidence was consistent for the absence of any significant associations between weight recurrence and carbohydrate, protein, and food patterns. However, the study findings for the association between weight recurrence and fat intakes was inconsistent, and an inverse association with diet quality was reported by just one cohort study. Therefore, two recommendations can be made: long-term diets post-bariatric surgery can be individualized with flexibility to macronutrient and food group composition due to lack of association (Grade C); and that a high diet quality can be encouraged in long-term post-surgery diets for reduction of weight recurrence (Grade D).

### Study findings for the dietary components not able to be graded

It was not within the scope of this review to have an in-depth analysis of the associations between energy intake and weight outcomes, due to not being included in the search term for systematic retrieval, and food patterns beyond core food group intakes due to not being consistently reported (e.g. specific food intakes like sandwiches, packaged foods or sweets that were defined differently across studies). These findings are presented as part of Table 2.

## Discussion

To our knowledge, the current review is the first to systematically synthesize existing literature reporting on associations between macronutrient composition, food patterns and weight outcomes post-bariatric surgery. Our review found that current evidence is related to the assessment of diet in distinct timeframes: ≤12 months, between 12- and 24-months and ≥24 months after surgery. Relationships between macronutrient intake and weight loss up to 24 months were inconclusive due to inconsistent findings between several studies of varying quality. Very few studies reported on food patterns, where the lack of studies and poor study design also contributed to inconclusive evidence. However, at 24 months and longer the evidence was consistent across several study findings for no association between macronutrient intake or food group pattern and weight loss, and between carbohydrate, protein and food group pattern and weight recurrence. An inverse association between weight recurrence and diet quality was reported from a single cohort study. Therefore, the existing body of evidence, overall, does not support a specific macronutrient composition or food pattern for optimal weight outcomes after bariatric surgery.

Previous research has suggested or attempted to establish the best macronutrient composition to support post-surgery weight loss [9, 40]. However, our review found the evidence base for associations with macronutrient composition and food pattern relied on less robust study designs and many contained confounding factors resulting in mixed results over different timeframes. The interpretation of association, therefore, was limited due to the lack of high-quality study designs. Despite this, the most notable phenomenon was that any relationship between macronutrients, food group pattern and weight outcomes, seemed to have lost significance after 24-months post-surgery, as studies that assessed dietary intakes after 24-months post-surgery found no associations whilst there was a mix of studies reporting the presence or absence of associations before 24 months [22, 23, 26, 28, 30, 31, 33, 35, 38, 39, 41–46, 50, 51, 55–57]. More well designed RCTs or prospective cohort studies are required to explore the potential associations between post-surgery weight outcomes with short- to medium- term dietary intakes (<24-months).

Although studies reporting on energy intake post-surgery were not systematically searched as part of this review, an inverse association with weight loss and positive association with weight recurrence was largely supported by those included studies that assessed energy intake [22–26, 28, 30, 31, 33, 35, 38–41, 43–46, 50, 51, 56]. Previous studies have debated the roles of long-term dietary restriction and food malabsorption in post-bariatric surgery weight outcomes [58–64]. However, there are known contributing factors that make it difficult to ascribe weight outcomes solely to energy intake, for example, the known prevalence of under-reporting dietary intakes among this population [59, 62, 63] and/or differences in energy balance as a result of individual basal requirements and physical activity levels [62, 65]. In Novais et al., only those with >50% excess weight loss reported an energy intake that was significantly lower than their estimated energy requirements. Furthermore, Benson-Davies et al. [59]. found participants who maintained their weight loss achieved a 2100-kilojoule deficit in energy balance (through a combination of lower energy intake and higher step counts) when compared to participants who experienced weight recurrence. Similarly, Forbush et al. suggested energy expenditure, rather than energy intake alone, to be a predictor of weight loss [62]. Given the limited reporting of physical activity levels in this population [65], energy balance may be an important focus for future research and practice to support long-term weight loss/maintenance post-bariatric surgery.

For many, the ultimate goal of achieving and sustaining weight losses after bariatric surgery is to improve the management of obesity-related complications. With the modest direct associations between post-surgical weight and diet composition found in this review, it may be sensible to place higher emphasis on exploring the impacts of diet composition on non-weight parameters of health, and specifically, those that are associated with the management of obesity-related complications. In the current review, studies that have reported non-weight clinical parameters mostly focused on the associations between body composition, quality of life and protein intakes [29, 37, 49, 53, 55], due to protein’s role in muscle mass maintenance, believed to be beneficial for weight loss maintenance. However, the overall evidence from the current review is mixed and does not support a high-protein diet for weight loss outcomes long-term post-surgery. This finding aligns with those of two previous systematic reviews focusing on the associations of protein intakes and body composition, which resulted in inconclusive findings [66, 67]. Larger and higher quality studies that place greater emphasis on obesity-related health parameters, in addition to weight, are required to explore how post-surgery diets may influence these parameters. Also, a recent study found no significant correlations between the extent of post-surgery weight loss and improvements in cardiovascular risk factors [68]. While this information was from a single study, it is apparent that future studies would benefit from placing additional emphasis on the relationship between post-surgery dietary intakes and clinical parameters, such as cardiometabolic health, in patients with or without weight non-response or recurrence at long-term post-bariatric surgery.

With the need to explore the relationships between obesity-related health parameters beyond weight, and in the absence of associations between weight non-response or recurrence with individual macronutrients and food groups, focusing on the overall quality of individuals’ diets may provide a more holistic approach to improving patients’ post-surgery eating pattern. In this review, Da Silva et al. was the only study retrieved that assessed diet quality in a systemic manner using an established tool, and suggested an ongoing weak association with weight re-occurrence after two years post-surgery [28]. Other identified studies that mentioned participants’ diet quality either did not use any established tool to measure diet quality [23, 33, 42], or did not adequately report participants’ dietary intakes [69–73], thus preventing comparisons across studies and, therefore, not included in the review as part of the data analyses for diet quality [23, 33, 42] or excluded for not meeting eligibility criteria [69–73]. Additionally, it is not known how useful or valid are established diet quality tools for use in this population [74, 75]. At present, existing research undertaken to derive and validate diet quality indices does not specifically consider bariatric surgery population [28, 70–76]. The potential changes in physiology and/or dietary needs after bariatric surgery may reduce the generalizability of diet quality indices from the general population to the bariatric surgery population. Without an accepted definition or measurement of high diet quality following bariatric surgery, it is not possible to compare the potential influence of diet quality on different obesity-related health parameters between studies and over-time. Future studies are needed to establish a consensus or criterion for the definition of a high-quality diet after bariatric surgery and to develop and validate a bariatric-specific diet quality measurement tool.

The differences in surgical procedure are often speculated as a contributor to different weight outcomes. Only one included study compared outcomes between different surgical types (SG versus RYGB), with no significant differences observed for weight outcomes and macronutrient composition [43]. Although findings for LAGB studies may not be generalizable across different surgery types, as they did not involve the resection of the gastro-intestinal tract, only 4 out of the 36 included studies involved post-LAGB patients. However, the differences in dietary intakes across surgical procedures were not the main interest of the current review. Since most of the included studies in this review reported single procedures only, and key findings did not differ across single or mixed surgeries or different surgery types, separate reporting of findings per surgery type was not attempted by this review. More studies may be needed to assess whether differences in surgical procedure can moderate the association between diet and post-surgery weight, and if so, whether such differences are significant enough to warrant separate dietary advice per surgery type. Another commonly speculated contributor to different weight outcomes is concomitant medications that may influence body weight (e.g. anti-depressants, corticoids, insulin) and the presence of T2DM, which may result in less weight loss than participants without T2DM [77]. In our review, only four of the included studies excluded participants taking these medications, but it was unclear whether the type(s) of medications considered in each study were the same [22, 23, 27, 28, 37]. Similarly, the rate of participants with insulin resistance or T2DM were only reported in a few studies and was often combined with other co-morbidities for assessment of the effects of number of co-morbidities on post-surgery weight [22, 25, 35, 36, 40]. The single study that assessed the potential impact of T2DM on excess weight loss reported no significant associations [25]. Another study reported higher rate of weight recurrence among those with insulin resistance, but acknowledged participants’ higher body fat percentage or BMI may have contributed in part to the results [22]. Future studies may need to clarify whether participants with medications or conditions that may influence participants’ weight are included in their statistical analysis, in order for higher confidence that the recorded weight changes are attributable to participants’ lifestyle.

The strengths of this review include the emphasis on long-term outcomes after bariatric surgery, and being the first review to systematically examine any potential associations between weight and dietary intakes after bariatric surgery. Limitations include the lack of inclusion of energy intake as a search criterion and the inability to discriminate the results by the type of surgery. The application, interpretation and generalization of results required subjective judgements to arrive at final recommendations. The retrieved studies were limited by a generally poor evidence base and poor consistency due to their methodological flaws and heterogeneity in study design, including different units of measure and different methods of assessment of association. Most studies did not account for dietary changes made before or after their dietary assessments, which may have played a role in weight status measured at time of study. Furthermore, the definitions used for post-surgery weight outcomes (weight non-response or weight recurrence) lacked standardization. These limitations align with the findings of a recent review that called for a research-derived definition of clinically significant post-surgery weight recurrence [78]. This lack of standardization limited comparisons between studies and the grading of evidence to support specific recommendations, particularly those assessing short-term dietary intakes (up to 24 months). The unknown contributors to weight outcomes such as physical activity, genetics and individual motivation, especially in studies with longer durations, were poorly reported within the included studies. Although the treatment of known confounders by the study authors were taken into consideration during risk of bias assessment, their interference on the potential clinical impact of the synthesized evidence was inevitable. Generalizability and applicability of studies to other bariatric contexts was also affected by individual study designs that may have incurred risk of selection bias, or contained inadequate information (e.g. adherence to prescribed diets, or dietary intakes prior to study intervention), to enable reproducibility of study outcomes to external populations. Moreover, the retrieved studies had a high proportion of female participants. Lastly, the results were prone to inherent limitations of dietary assessment methodologies such as recall bias and under-reporting. While there are standardized methods for excluding implausible reporting [79], only two studies in our review attempted to address the known risk of under-reporting by comparing two methods of self-report (a 72 h recall versus a three day food record) to evaluate compliance to prescribed diets, which reported high compliance and similar energy intakes [53, 54]. The remaining studies simply acknowledged this limitation, but did not identify nor exclude potential under-reporters [24–52, 55–57]. Even though one study hoped to use the Goldberg cut-offs to assess implausible intakes among their participants within 12-months post-surgery, said method assumes weight stability and was deemed unsuitable for use for patients who had yet to reach weight maintenance stage after surgery [29]. Importantly, there are no standardized energy intake cut-offs for this population to assist with assessment of implausible intakes, which further complicated the exclusion of implausible reporters. As this limitation was discussed by some of the included studies as a potential contributor to the lack of association found between dietary intakes and weight outcomes, future studies on patients post-bariatric surgery may benefit from including the assessment of implausible intakes using estimated energy requirements, supplementing food diaries with weighed food records or photographed pictures of participants’ meals, adjusting for total energy intake based on two methods (e.g. a food frequency questionnaire and three 24 h food recalls), or other standardized methods, to help improve accuracy of dietary assessments.

## Conclusion

In view of the overall finding that there is a lack of evidence to support the strong association of any particular diet composition or pattern with weight loss or recurrence, it is not unreasonable to suggest that long-term dietary advice post-bariatric surgery can be individualized with flexibility as to the macronutrient and food composition, or that a focus on diet quality may be beneficial for the reduction of weight recurrence over the longer term. However, these recommendations should be taken with care until higher quality studies further confirm and strengthen these statements. Well-designed prospective trials with standardized reporting and monitoring of dietary intakes including energy intake/expenditure, coupled with the considerations of potential moderators of weight, such as the type of surgery, may be beneficial to help clarify the relationships between weight outcomes and dietary intakes.

## Supplementary information

Supplementary Material 1

## Data Availability

Data sharing is not applicable to this article as no datasets were generated or analyzed during the current study. The search strategy of the current review is included as Supplementary Material 1.
