# Supplementary material for: Associations between diet composition, dietary pattern, and weight outcomes after bariatric surgery: a systematic review
Source: Int J Obes (Lond). 2023 Jul 6;47(9):764–90. doi: 10.1038/s41366-023-01333-1 (PMC10439005; doi:10.1038/s41366-023-01333-1)
Supplement: Supplementary file 1 — Supplementary Material 1 [file 41366_2023_1333_MOESM1_ESM.docx]

# Supplementary Material 1 – Search terms

## Medline

| **Search number** | **Search Term** | **Limitors** |
| --- | --- | --- |
| S1 | (post OR after OR following) w3 “Bariatric surgery” OR ”weight loss surgery” OR "gastric bypass” OR ”gastric sleeve” OR ”sleeve  gastrectomy” | Abstract only  English only, Human only |
| S2 | (MH "Bariatric Surgery+") | English only, Human only |
| S3 | S1 OR S2 | - |
| S4 | Diet* OR food OR nutrition* OR eat* OR macronutrient* OR ‘postoperative diet’ | Abstract only |
| S5 | (MH "Nutritional Requirements+") OR (MH "Portion Size") OR (MH "Serving Size") OR (MH "Energy Intake+") OR (MH "Diet, Healthy") OR (MH "Diet, High-Protein+") OR (MH "Diet, Reducing") OR (MH "Diet, FatRestricted") OR (MH "Diet, CarbohydrateRestricted+") | - |
| S6 | S4 OR S5 | - |
| S7 | S3 AND S6 | English only, Human only |

## CINAHL

| **Search number** | **Search Term** | **Limitors** |
| --- | --- | --- |
| S1 | (post OR after OR following) w3 “Bariatric surgery” OR ”weight loss surgery” OR "gastric bypass” OR ”gastric sleeve” OR ”sleeve  gastrectomy” | Abstract only |
| S2 | (MH "Bariatric Surgery+") | - |
| S3 | S1 OR S2 | - |
| S4 | Diet* OR food OR nutrition* OR eat* OR macronutrient* OR ‘postoperative diet’ | Abstract only |
| S5 | (MH "Diet, Fat Restricted") OR (MH "Diet, Low Carbohydrate") OR (MH "Diet, Reducing") OR (MH "Diet, High Protein") OR (MH "Food Intake+") OR (MH "Energy Intake") OR (MH "Portion Size") OR (MH "Nutritional Requirements+") | - |
| S6 | S4 OR S5 | - |
| S7 | S3 AND S6 | English only, Human only |

## Cochrane

| **Search number** | **Search Term** | **Limitors** |
| --- | --- | --- |
| S1 | (post OR after OR following) NEAR/3 ("bariatric surgery" OR "weight loss surgery" OR "gastric bypass" OR "gastric sleeve" OR "sleeve gastrectomy") in Record Title AND Diet* OR food OR nutrition* OR eat* OR macronutrient* OR "postoperative diet" in Title Abstract Keyword - (Word variations have been searched) | - |

## Embase

| **Search number** | **Search Term** | **Limitors** |
| --- | --- | --- |
| S1 | (post OR after OR following) near/3 “Bariatric surgery” OR ”weight loss surgery” OR "gastric bypass” OR ”gastric sleeve” OR ”sleeve  gastrectomy” | Title only |
| S2 | 'bariatric surgery'/exp | - |
| S3 | #1 OR #2 |  |
| S4 | Diet* OR food OR nutrition* OR eat* OR macronutrient* OR ‘postoperative diet’ | Title/Abstract only |
| S5 | 'diet composition'/exp OR 'dietary intake'/exp OR 'dietary pattern'/exp OR 'food intake'/exp OR 'nutritional requirement'/exp OR 'portion size'/exp OR 'protein diet'/exp OR 'caloric restriction'/exp OR 'low fat diet'/exp OR 'low carbohydrate diet'/exp | - |
| S6 | #4 OR #5 | - |
| S7 | #3 AND #6 AND [humans]/lim AND [english]/lim NOT pregnan* NOT breastfeed* NOT child* NOT adolescen* NOT p$ediatric AND ([article]/lim OR [article in press]/lim OR [conference paper]/lim OR [conference review]/lim OR [data papers]/lim OR [erratum]/lim OR [review]/lim) AND [embase]/lim NOT ([embase]/lim AND [medline]/lim) | English only, Human only, Embase only, article/article in press/conference paper/conference review/data paper/erratum/review only |

## Scopus

| **Search number** | **Search Term** | **Limitors** |
| --- | --- | --- |
| S1 | ( TITLE-ABS-KEY ( ( post OR after OR following ) W/3 "Bariatric surgery" OR "weight loss surgery" OR "gastric bypass" OR "gastric sleeve" OR "sleeve gastrectomy" ) ) AND ( TITLE-ABS-KEY ( diet* OR food OR nutrition* OR eat* OR macronutrient* OR 'postoperative AND diet' ) ) AND NOT ( animals ) AND NOT ( animals AND humans ) AND ( LIMIT-TO ( LANGUAGE , "English" ) ) | Title, abstract or keyword  English, human only |
